# Bismuth Confinement: A Strategy for Low Resistance and Good Thermal Endurance of Integrated Contacts to MoS_2_

**DOI:** 10.1021/acsnano.5c19217

**Published:** 2026-02-24

**Authors:** Wen-Chia Wu, Terry Y. T. Hung, Fu-Kuo Hsueh, Bo-Heng Liu, Wei-Sheng Yun, Yun-Yan Chung, Yu-Ching Wang, Ze-Rui Lin, Meng-Zhan Li, Zih-Siang Jian, Szu-Huan Hsu, Jian-Chen Tsai, Jyun-Hong Chen, Chien-Wei Chen, Yiming Li, Wen-Hao Chang, Wei-Yen Woon, Chi-Chung Kei, Tuo-Hung Hou, Chao-Ching Cheng, Iuliana P. Radu, Chao-Hsin Chien

**Affiliations:** † Institute of Electronics, 34914 National Yang Ming Chiao Tung University, Hsinchu 30010, Taiwan; ‡ Corporate Research, Taiwan Semiconductor Manufacturing Company, Hsinchu 30075, Taiwan; § Taiwan Semiconductor Research Institute, Hsinchu 300091, Taiwan; ∥ National Center for Instrumentation Research, 63353 National Institutes of Applied Research, Hsinchu 30076, Taiwan; ⊥ Department of Electrophysics, National Yang Ming Chiao Tung University, Hsinchu 30010, Taiwan; # Parallel and Scientific Computing Laboratory, National Yang Ming Chiao Tung University, Hsinchu 30010, Taiwan; ¶ Institute of Communications Engineering, National Yang Ming Chiao Tung University, Hsinchu 30010, Taiwan

**Keywords:** two-dimensional semiconductors, semimetal contacts, contact resistance, back-end-of-line (BEOL) integration, monolithic 3D integration, thermal stability

## Abstract

Two-dimensional (2D) transition metal dichalcogenides (TMDs) are preponderant candidates for advanced nanoelectronics owing to their atomically thin body, which could enable excellent electrostatic control. High current density NMOS transistors have been demonstrated with semimetal Bi and Sb contacts. Both these semimetals have low melting temperatures and thus limit their integration flow compatibility with the modern integrated circuit technologies. Integration of those metal layers requires exposing the transistors to 400 °C H_2_ environment for extended periods of time. Using a Bi confinement strategy through AlO_*x*_ and/or TiN barriers, *R*_C_ values below 200 Ω·μm are demonstrated while preserving device performance after forming gas annealing at 400 °C for up to 10 min; this finding is validated through the characterizations of multiple devices. Furthermore, by using fab-like SiO_2_ trench structures with TiN barrier and W plug in addition to the Bi, we confirm the thermal stability of the structures. The thermal stability is found to be good at 400 °C under several process environments, such as N_2_, forming gas, and vacuum. Cross-sectional TEM and EDX confirm that Bi contacts remain fully confined without diffusion or outgassing, establishing the process compatibility of this confinement method. This study establishes a fab-compatible confinement strategy that enables low *R*_C_ monolayer MoS_2_ transistors while maintaining thermal robustness.

## Graphical abstract

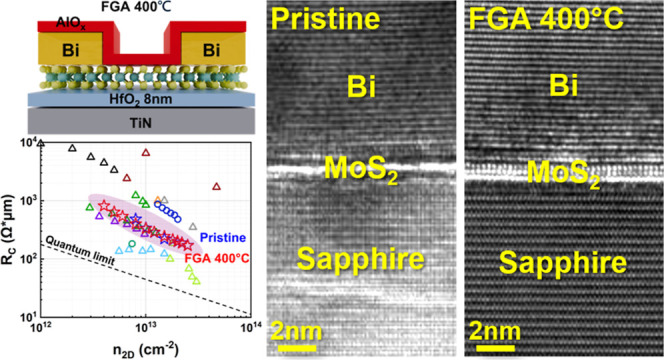

## Introduction

Two-dimensional (2D) semiconductors, such as transition metal dichalcogenides (TMDs), have attracted extensive attention as promising channel materials for future nanoelectronic devices. Their atomically thin body enables excellent electrostatic control at deeply channel length-scaled dimensions,^1–4^ positioning them as predominant candidates for further transistor device scaling.^5–8^ Not only the gate length scaling, but also the potential for contact length shrinkage has been demonstrated.^9,10^ These unique attributes put forth 2D semiconductors as highly attractive materials for transistor scaling and either in addition to Si through back-end integration schemes^11–19^ or replacing Si.^20^

While promising projections have been accompanied by promising lab results, there remain only limited studies addressing how to integrate 2D transistors into the process flows for the contemporary integrated circuit manufacturing.^21–23^ The challenges are manifold, which come from (1) channel formation, (2) gate stack, (3) contacts, but also from (4) consistent translation of lab device learning into fab flows. First, 2D TMD material growth itself remains a key hurdle, as high-quality wafer-scale synthesis must be achieved in a fab-compatible and economical way.^24–27^ Similarly, solutions for low-defect gate stacks prove to be challenging.^28–30^ This paper, however, will focus only on the third and fourth challenges, those of contact resistance and finding fab-compatible approaches to low contact resistance. The contact challenge and translation to fab-like flows are common to all new channel materials.

Best performance, most scaled lab NMOS devices have been reported with a monolayer MoS_2_ channel. Their fab counterparts are far lagging in performance, limited by *R*_C_ from edge-only contact.^23^ In the case of monolayer MoS_2_, achieving low contact resistance (*R*_C_) typically requires semimetals^9,10,31–35^ such as bismuth (Bi) or antimony (Sb), which provide van der Waals–like interfaces and favorable band alignment for electron injection. However, these low-melting-point semimetals are thermally unstable, prone to agglomeration or evaporation, or sublimation during postprocessing, leading to catastrophic degradation.^36^ Using refractory metals with high melting points while offering superior thermal stability induces prohibitively high *R*_C_ (>1 kΩ·μm), undermining device performance.^37–46^ This trade-off between contact resistance and contact material thermal stability has thus far prevented translation of key lab learnings to fab-like integration approaches.

Addressing this challenge is critical, as contacts remain the bottleneck in 2D transistor integration. Without a viable scheme to simultaneously ensure low *R*_C_ and BEOL stability, the deployment of 2D materials in industry-relevant process flows remained elusive. While prior reports have explored semimetal contacts for high-performance 2D devices, no work has demonstrated their ability to endure BEOL formation or BEOL-compatibility through annealing while preserving contact integrity and low contact resistance. In this work, we therefore specifically resolve the contact thermal stability problem, as it represents one of the most pressing barriers toward practical integration of 2D transistors. A second challenge associated with the proposed contacting scheme, that of a dielectric etch process with high selectivity toward the 2D channel is still to be solved and will be addressed in subsequent publications.

In this publication, we demonstrate that a confinement strategy enables semimetal bismuth contacts to achieve low contact resistance (<200 Ω·μm) while maintaining robustness under BEOL-like annealing (400 °C, forming gas condition). Unlike prior studies^9,10,31–35^ that either focus on low-*R*_C_ contacts without thermal stability or stable contacts with prohibitively high resistance,^37–45^ our approach uniquely achieves this trade-off. Beyond the device-level demonstration, we further show compatibility with a fab-like approach by constructing SiO_2_ trench structures within which the Bi is further confined by a TiN barrier and W plug, mimicking realistic transistor contact process flows.^47–50^ Cross-sectional TEM and EDX analyses confirm that Bi contacts remain fully confined without diffusion or outgassing even under extended BEOL annealing. Together, these results highlight a scalable and process-compatible strategy for integrating contact to 2D transistors into transistor fabrication.

## Results and Discussion

Modern chips, in addition to high-performance transistors, require multiple levels of metal interconnect to link those individual transistors into circuits and to the world outside the chip (Figure 1a). Metal interconnect will be referred to as BEOL (back-end of line). Fabricating these BEOL layers requires thermal budgets of at least 350–400 °C while the environment can be various dilutions of H_2_ or N_2_. Typically, lab devices built through metal-contact lift-off do not test for compatibility of the channels and contact materials with subsequent fabrication of the BEOL. The thermal budget, however, is critical because in monolithic integration, any instability in the contact layers during annealing could compromise the entire stack.

**Figure 1:**
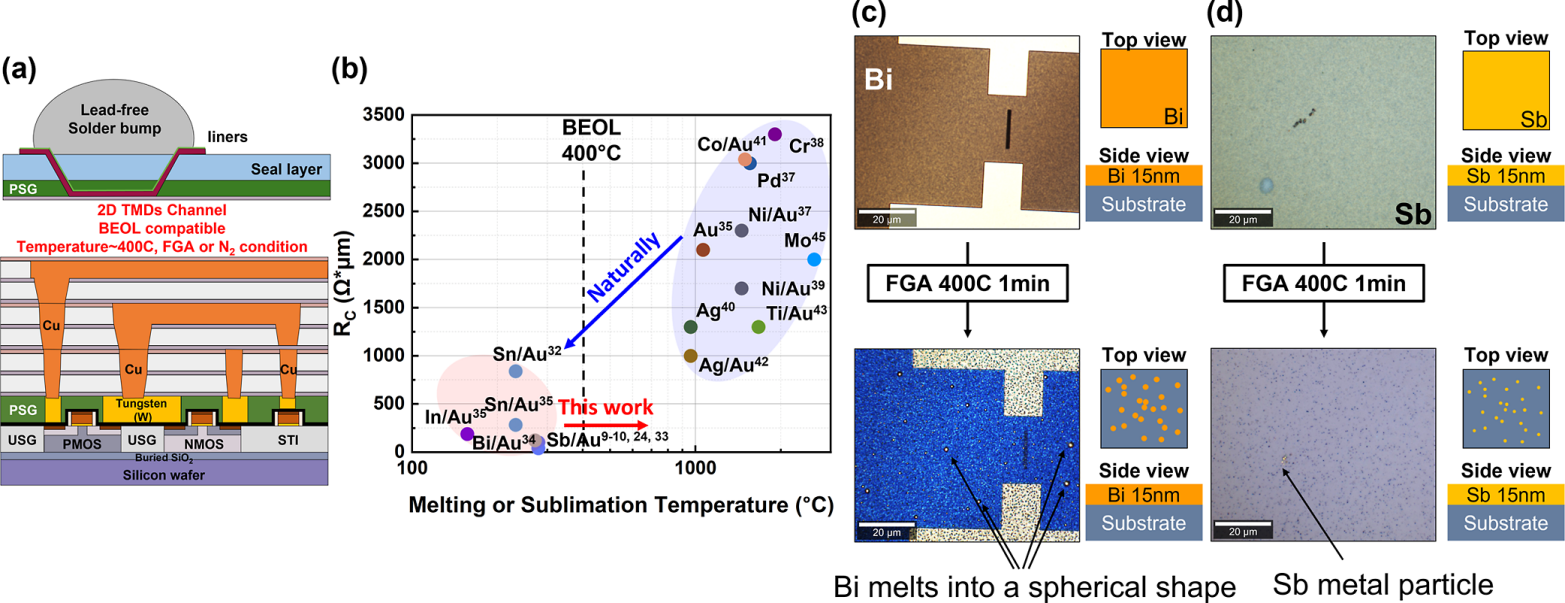
Key challenges for low *R*_C_ integrated contact to transistor with 2D material channel. (a) Schematic illustration of transistors with 2D materials channel and the subsequent interconnect metal layers. Devices must retain high performance and thermal stability during back-end processing (≤400 °C in forming gas). (b) Conceptual trade-off between contact resistance (*R*_C_) and BEOL robustness: refractory metals, while having a high melting temperature, typically have high *R*_C_ to 2D channel, higher than 1 kΩ·μm. Semimetals such as Bi and Sb provide ultralow *R*_C_ but are thermally unstable. (c) Optical images of Bi contacts before and after annealing at 400 °C for 1 min, showing melting and dewetting into droplets. (d) Optical images of Sb contacts under identical annealing conditions, where Sb rapidly sublimates and disappears. These results highlight the intrinsic fragility of semimetal contacts under BEOL stress, motivating the confinement strategy explored in this work.

Current literature has reported several contact schemes for 2D TMD NFETs, but none fully satisfy the dual requirements of low contact resistance (*R*_C_) and BEOL-compatible thermal robustness.^36,51–53^ High–melting-point refractory metals can withstand higher temperature annealing but generally induce *R*_C_ values above 1 kΩ·μm, far too large for high-performance device targets. In contrast, semimetals such as Bi and Sb provide *R*_C_ values closer to the quantum limit, demonstrating excellent injection properties. See Figure 1b for a summary of reported contact resistance as a function of the melting or sublimation temperature of the contact material. The inherently low melting or sublimation points (<400 °C) of the semimetals render them unstable under BEOL conditions; indeed, Sb readily vaporizes even under mild pressure, and Bi tends to agglomerate or melt during standard annealing. Figure 1c,d illustrate these instabilities: Bi contacts melt and form spherical droplets, while Sb rapidly disappears after just 1 min of annealing at 400 °C in forming gas, underscoring their thermal fragility.

To overcome this long-standing trade-off, we introduce a capping-layer confinement strategy designed to stabilize low–critical temperature contacts without sacrificing their inherently low *R*_C_. By employing this approach, we aim to enable 2D transistors that combine state-of-the-art contact resistance with BEOL-compatible robustness, establishing a pathway toward integration in monolithic 3D structures.

Since the melting temperature of Bi and Sb is relatively low, their exposure to annealing environments can be catastrophic.^36^ However, with a strategy of placing Bi and Sb in confined structures formed by dielectrics or other metals, the structural integrity of the contacts and low *R*_C_ can be maintained nicely, as illustrated in Figure 1c,d. In the following figures, we show that TiN, SiO_*x*_ and AlO_*x*_ can act as effective diffusion barriers.

Antimony Sb films deposited on 90 nm SiO_2_ and capped by low-temperature AlO_*x*_ initially appear rough and discontinuous, forming spherical islands (Figure 2a) as the Sb has limited wettability on SiO_2_. Upon stepwise annealing from up to 800 °C in forming gas (N_2_/H_2_), AlO_*x*_-capped Sb films remain confined well above their sublimation and melting points. TEM images confirm recrystallization of the Sb films without any sign of outgassing. Ostensibly, the semimetal transits toward a flattened, crystalline morphology, underscoring the confinement effect of AlO_*x*_. Compared to the unstable as-deposited islands, the annealed and confined overmelting-point Sb layers exhibit smoother, more continuous surfaces, which could enable lower *R*_C_ variability.

**Figure 2:**
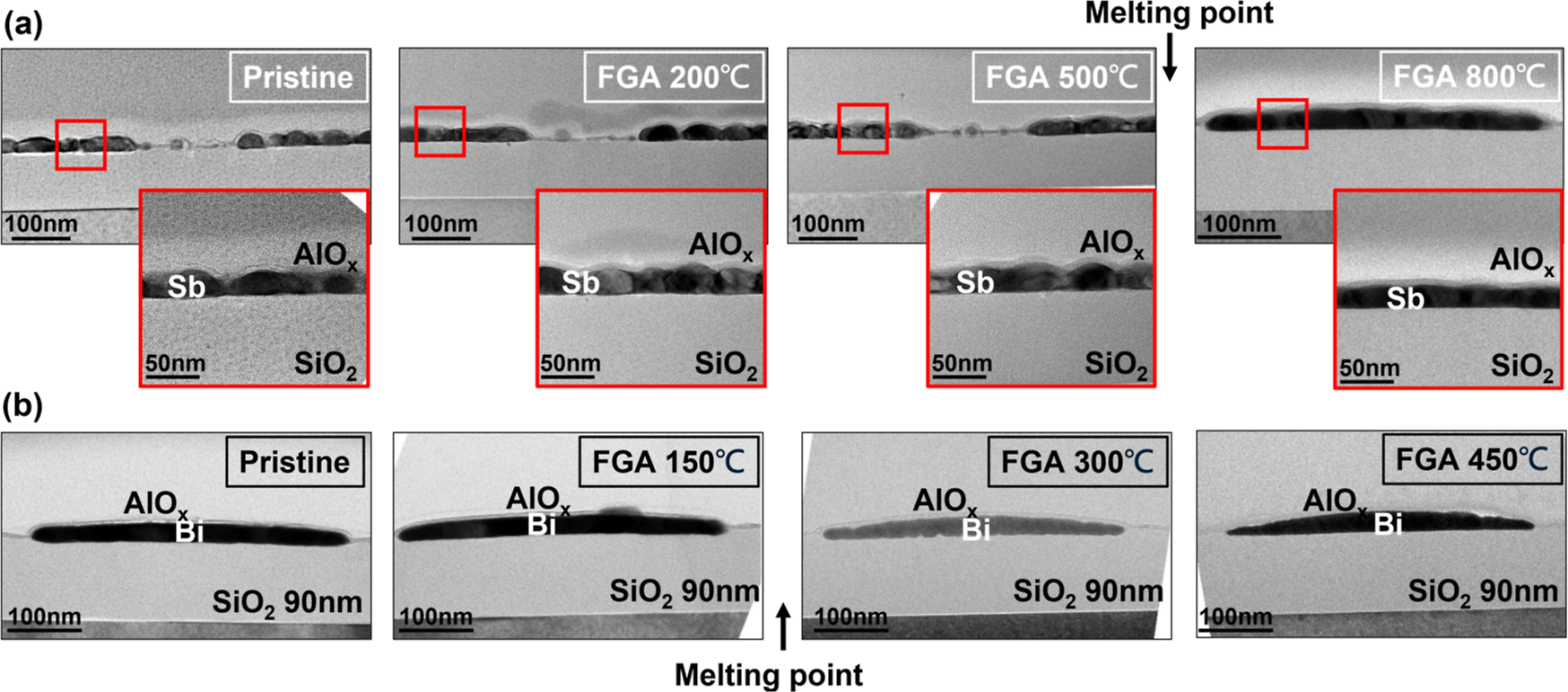
Confinement stabilizes low-melting-point semimetal contacts under BEOL processing conditions. (a) Antimony films deposited on SiO_2_ and capped with low-temperature ALD AlO_*x*_. As-deposited Sb appears rough and discontinuous, forming spherical islands, but after stepwise annealing up to 800 °C in forming gas, the morphology becomes flattened and crystalline, with no evidence of outgassing or sublimation. TEM images confirm confinement beyond the Sb melting point (671 °C). (b) Bismuth contacts with AlO_*x*_ capping shows similar stabilization, enduring stepwise annealing up to 450 °C—well above the BEOL thermal budget—without melting, agglomeration, or vaporization. Together, these results demonstrate that AlO_*x*_ effectively confines Bi and Sb, enabling the layers to withstand 400 °C forming gas annealing without morphological degradation.

A similar stabilization effect is observed for Bi. Without confinement, Bi readily melts at ∼273 °C, far below the BEOL target, precluding its use in practical integration. However, when encapsulated by AlO_*x*_, Bi contacts endure stepwise annealing up to 450 °C—exceeding the BEOL thermal budget—without agglomeration, or visible vaporization (Figure 2b). This confirms that even low–melting temperature contacts can be preserved under BEOL conditions when properly confined. Further detailed TEM analysis and study of the area dependence are included in Figures S1–S3.

Figure 2 establishes the concept that capping the semimetals with a material that limits their diffusion stabilizes them well beyond their intrinsic thermal limits, enabling 400 °C forming gas annealing without outgassing or morphological failure. These results establish AlO_*x*_ as a practical proxy for conventional TiN/TaN barriers in monolithic 3D integration, offering a simple and effective approach to obtain thermally robust contacts.

To evaluate whether the AlO_*x*_ confinement strategy extends beyond material stabilization to actual device performance, we fabricated monolayer MoS_2_ transistors with Bi contacts with AlO_*x*_ capping and subjected them to BEOL-compatible annealing. The device structure (Figure 3a) consists of a monolayer CVD MoS_2_ channel on 8 nm HfO_2_ gate dielectric (EOT–2 nm), with a TiN back gate metal. The Bi contacts are confined between 90 °C ALD AlO_*x*_, MoS_2_, and HfO_*x*_ (process flow shown in Figure S4). Cross-sectional TEM (Figure 3b) confirms that the Bi morphology remains intact after forming gas annealing (400 °C, 6 Torr, 10 min), verifying the effectiveness of the confining structure, where not only the AlO_*x*_ but also the monolayer MoS_2_ limits its diffusion. Additional high-resolution TEM analyses further corroborating the effectiveness of the confinement strategy on monolayer MoS_2_ are provided in Figures S5 and S6.

**Figure 3:**
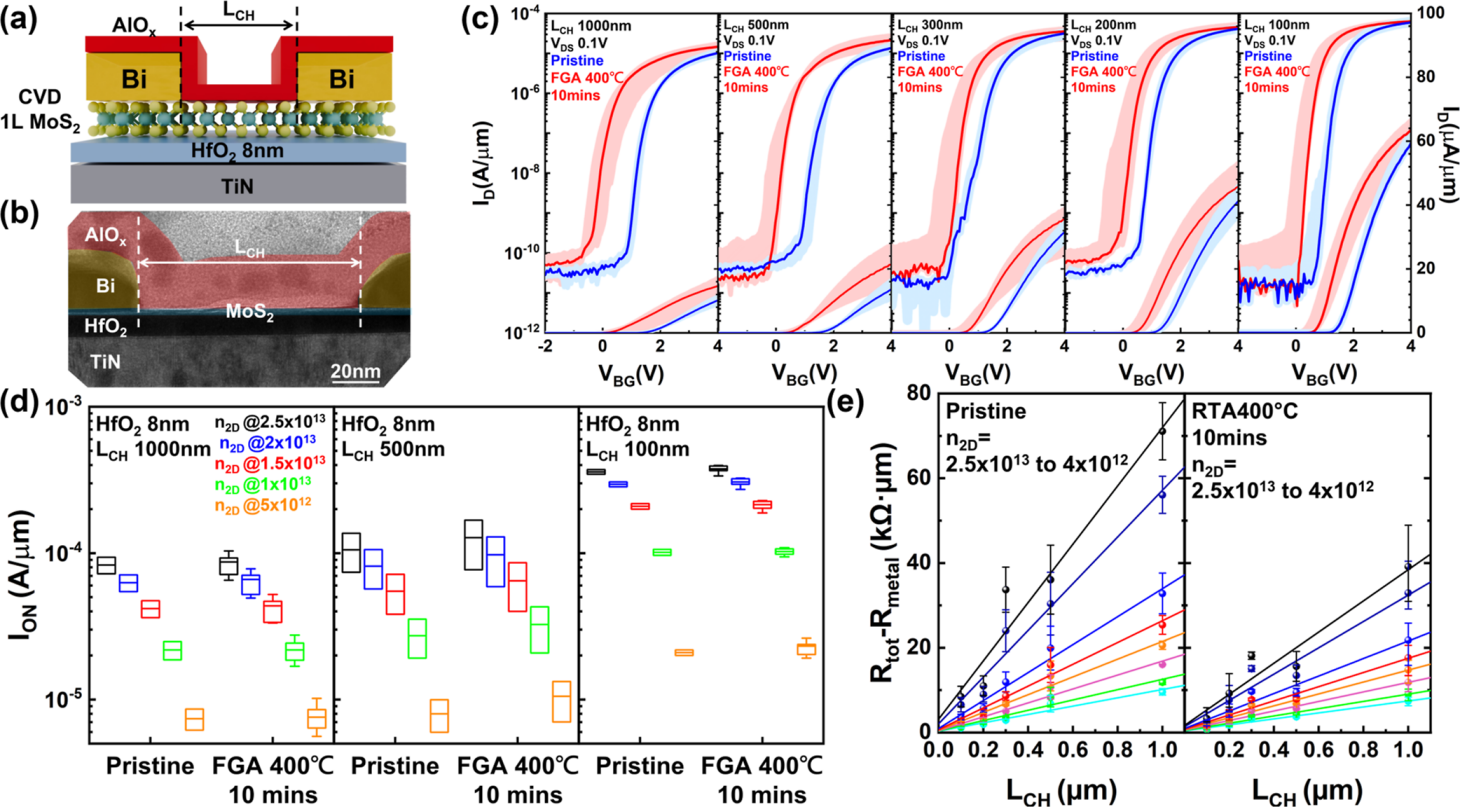
Device-level demonstration of Bi/AlO_*x*_ contacts after annealing as needed for BEOL processing. (a) Schematic of the MoS_2_ transistor structure: monolayer CVD-grown MoS_2_ on 8 nm HfO_2_ (EOT–2 nm) with a TiN back gate, contacted by Bi electrodes fully encapsulated with low-temperature ALD AlO_*x*_. (b) Cross-sectional TEM after 400 °C forming gas annealing (10 min) confirms that Bi morphology remains unaltered and well-confined by AlO_*x*_. (c) Transfer characteristics at V_DS_ = 0.1 V for devices with channel lengths of 1000–100 nm, showing nearly ohmic injection before (Blue) and after (Red) annealing. After annealing, devices exhibit enhanced drive current accompanied by a consistent negative V_TH_ shift across channel lengths. (d) On-current comparison at V_DS_ = 1 V under matched carrier concentrations (2.5 × 10^13^ to 5 × 10^12^ cm^-2^), demonstrating that current levels are comparable before and after annealing. (e) Statistical TLM analysis across multiple channel lengths, with metal line access resistance subtracted, yields *R*_C_ consistently below 200 Ω·μm both before and after annealing. Intrinsic mobility shows minor improvement from 27 to 32 cm^2^ V^–1^ s^–1^. These results confirm that confined Bi contacts retain low *R*_C_ and high performance under BEOL annealing, translating confinement stability from materials to functional devices.

Electrical characterization before and after annealing confirms the robustness of this scheme. Transfer curves at V_DS_ = 0.1 V for devices with L_CH_ = 100–1000 nm, with the median curve colored in a darker color, are presented in Figure 3c. The electrical characterization shows that before and after FGA, devices exhibit nearly ohmic injection, with I_ON_ over 60 μA/μm at L_CH_ = 100 nm. After annealing, the devices display enhanced maximum current density induced by negative threshold voltage V_TH_ shift. This is a consistent fixed positive charge in AlO_*x*,_ which induces additional electrons into the channel as reported elsewhere.^54–56^ Subthreshold swing remains largely unaffected (see Figure S7), indicating negligible interface degradation. Figures S8–S11 provide additional statistical comparisons of device characteristics before and after AlO_*x*_ capping and FGA at 400 °C, including on-current, threshold voltage, and hysteresis.

To account for threshold voltage shifts and allow a fair comparison, device behavior is analyzed at the same carrier density (Figure 3d). Box plots are used to represent all current values across all devices at a given channel length. The on-current at V_DS_ = 1 V across representative channel lengths (100,500, and 1000 nm) and carrier densities (2.5 × 10^13^ to 5 × 10^12^ cm^–2^) has similar values before and after annealing. Whole channel length comparison is shown in Figure S9d. The Bi confinement scheme is thus confirmed to enable undisturbed electrical performance upon 400 °C forming gas annealing, and hence its compatibility with BEOL formation.

The direct comparison of the current densities reported here to those previously published is limited by the intrinsic resistance of Bi metal lines. Following deposition and layout optimization, the Bi metal-line resistance is reduced to ∼1 kΩ (details of the metal-line resistance optimization, layout design, and statistical extraction are provided in Figure S12), arising from using long metal lines as required by the subsequent materials characterization performed on the devices. This access resistance would be much reduced in a fully integrated vehicle, as depicted below. To reasonably compare with our previous data, we used the transfer length method (TLM) analysis^57^ over multiple channel lengths with multiple devices at each channel length, subtracting the corresponding Bi resistance contribution. The extracted contact resistance (Figure 3e) remains consistent with the sub-200 Ω·μm regime both before and after forming gas annealing, demonstrating the stability of the confined Bi contacts. The intrinsic mobility derived from sheet resistance shows a slight improvement from 27 to 32 cm^2^ V^–1^ s^–1^ (see Figure S13), suggesting that annealing might reduce scattering via partial defect passivation.^54,55^

To assess the significance of the Bi confinement strategy, we benchmarked the contact resistance (*R*_C_) and maximum on-current (I_ON_) of Bi/AlO_*x*_ devices against reported 2D contacts before and after 400 °C annealing. *R*_C_ and mobility were extracted using the TLM method and subtracting line resistance as above before and after BEOL-compatible forming gas annealing (400 °C, 10 min), allowing direct quantification of contact and channel contributions and we do further perform numerical device TCAD simulations as our previous literature model.^9,58,59^ The simulation of the MoS_2_ devices before and after FGA annealing to extract the Schottky barrier height (SBH) and the van der Waals (vdW) gap. The extracted SBH is 70 meV and the vdW gap is equal to 2 Å at the MoS_2_/Bi contact, the result shows nearly ohmic contact similar to our TLM extraction data and also with narrow vdW gap before and after FGA annealing, which means Bi contact did not degrade through FGA treatment (Additional simulation details are provided in Figure S14.).

Figure 4a plots *R*_C_ extracted from the devices in Figure 3e (blue and red hollow-star symbols) alongside literature references. The *R*_C_ values of Bi/AlO_*x*_ remain consistently lower or comparable to 200 Ω·μm across carrier concentrations, with minimal change before and after annealing. Figure S15 presents the statistical TLM analysis (total resistance versus channel length) under the three processing conditions, together with the extracted contact resistance distributions. The data here is comparable to the best-performing contacts (triangle and circle symbols).^9,10,32–35,39,42,54,60–64,74^ Figure 4b benchmarks I_ON_ versus channel length at V_DS_ = 1 V.^9,32–35,39,42,54,61,63–74^ The devices reported here (symbols in blue and red) display strong drive current both before and after annealing, with I_ON_ values at L_CH_ = 100 nm ranking among the highest reported in the literature. As described above, residual Bi line resistance is non-negligible in raw data (triangle markers). Upon line resistance subtraction, the star marker data show Ion exceeding one mA/μm at V_DS_ = 1 V. Upon annealing, maximum current increases markedly, which is attributed to the negative V_TH_ shift mentioned above. The devices exhibit high current density after 400 °C annealing, demonstrating exceptional thermal stability among integrated 2D contacts.

**Figure 4:**
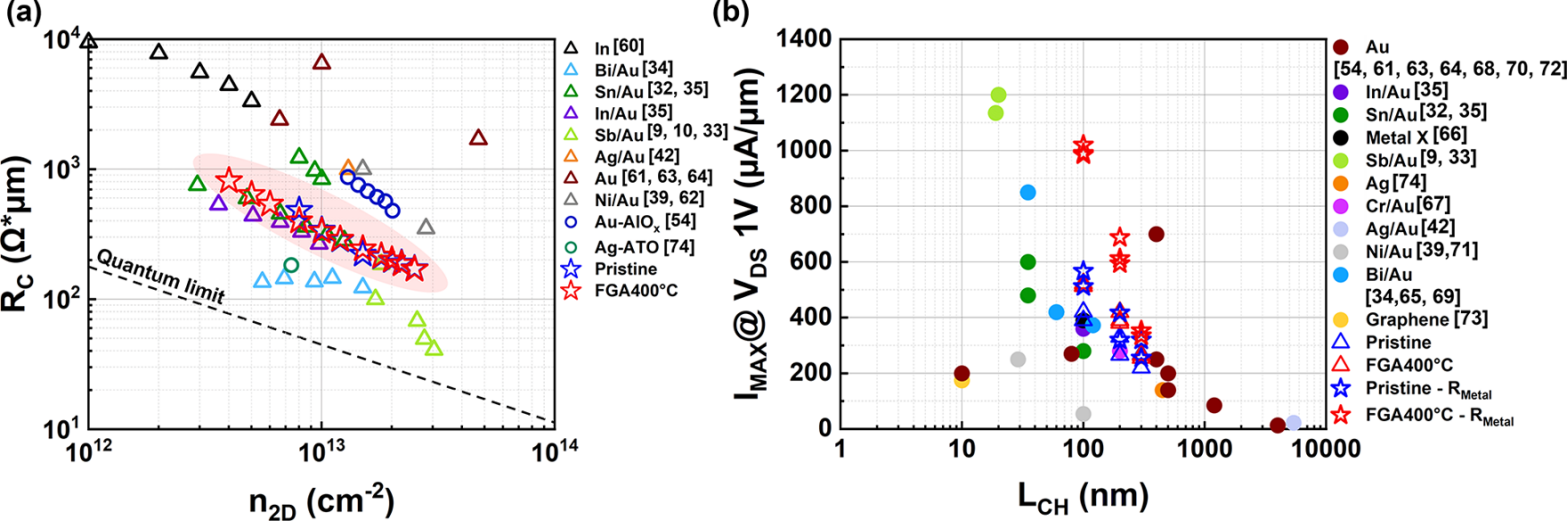
Comparison of confined Bi contacts before and after BEOL annealing against state-of-the-art 2D transistor contacts. (a) Contact resistance (*R*_C_) extracted by TLM before and after 400 °C, 10 min forming gas annealing, compared with literature reports. Confined Bi devices before (blue hollow stars) and after annealing (red hollow stars) consistently exhibit *R*_C_ values in the sub-200 Ω·μm regime, with minimal variation across carrier concentrations, comparable to the lowest-resistance contacts reported for 2D semiconductors. Reference values for semimetal contacts (triangles) and doped-metal contacts (circles) in monolayer MoS_2_ are included for comparison. (b) Benchmark of maximum on-current (I_ON_) versus channel length at V_DS_ = 1 V. Confined Bi devices (symbols in blue and red) retain strong drive currents after annealing, with L_CH_ = 100 nm devices showing some of the highest I_ON_ values reported to date for monolayer MoS_2_ channel. Subtracting Bi line resistance (see S.I.) yields an I_ON_ exceeding 1 mA/μm at V_DS_ = 1 V, underscoring the intrinsic capability of this contact strategy to enable high current density. Together, these benchmarks establish that this contact strategy simultaneously achieves record-low *R*_C_ and best-inclass I_ON_ while preserving stability under BEOL thermal budget and processing conditions.

Having established that confining Bi allows for maintaining low contact resistance and current density in transistors during BEOL mimicking annealing, we now evaluate its use in a fab-relevant contact scheme. The Bi is placed at the bottom of contact holes formed in the dielectric, with SiO_2_ used here as the dielectric. In an integrated flow, the contact holes are opened by etching the dielectric, stopping at the semiconductor channel. To reduce access resistance and suppress Bi diffusion, a TiN layer is implemented on top of the Bi. TiN and TaN barriers are commonly used in conjunction with interconnect metals such as Cu. The barriers reduce Cu diffusion in dielectrics upon thermal stress and limit Cu electromigration upon electrical stress. In the following, we demonstrate that a TiN strategy can be used to confine Bi at temperatures above its melting point.

A simplified test structure of the 3D contact hole geometry is built as detailed in Figure 5. A selective etch process capable of etching SiO_2_ while stopping on 2D TMDs^21^ is still one of the grand challenges for the adoption of 2D semiconductors. Thus, the simplified test structure of the contact only maintains the 3D geometry—focusing specifically on the contact/barrier stack integrity, rather than channel transport. As shown in Figure 5a, a SiO_2_ layer ∼100 nm thick was deposited on Si and patterned using dry etch to define trenches less than 40 nm wide. Bi is deposited via PVD in a deposition process similar to that enabling low-*R*_C_ contact metal for 2D devices (Figure 5b) through lift-off. Minor Bi deposition on the sidewall is observed due to the line-of-sight nature of PVD, consistent with expectations for this geometry. A conformal TiN barrier is introduced by 200 °C ALD to encapsulate the Bi contact (Figure 5c), forming a barrier-confined structure within the SiO_2_ trench; however, unlike the device demonstrated above, the barrier here is a good electron conductor. The Bi/TiN stack undergoes a BEOL-like thermal treatment in Figure 5d (400 °C, 10 min, forming gas). The subsequent cross-sectional TEM confirms that Bi remained intact, with no signs of outgassing, void formation, or morphological degradation. These results follow the material-level stability observed with AlO_*x*_-capped planar stacks (Figures 1 and 2), now extended to a realistic contact configuration with conductive capping. In the final fabrication step, the contact holes are filled with W using PVD as in a standard metal plug integration flow (Figure 5e). Central voids in the W plug arise from the nonconformal nature of unbiased PVD and are known to be mitigated by applying bias sputtering, chemical vapor deposition, or ALD-based gap fill strategies in practical flows.

**Figure 5:**
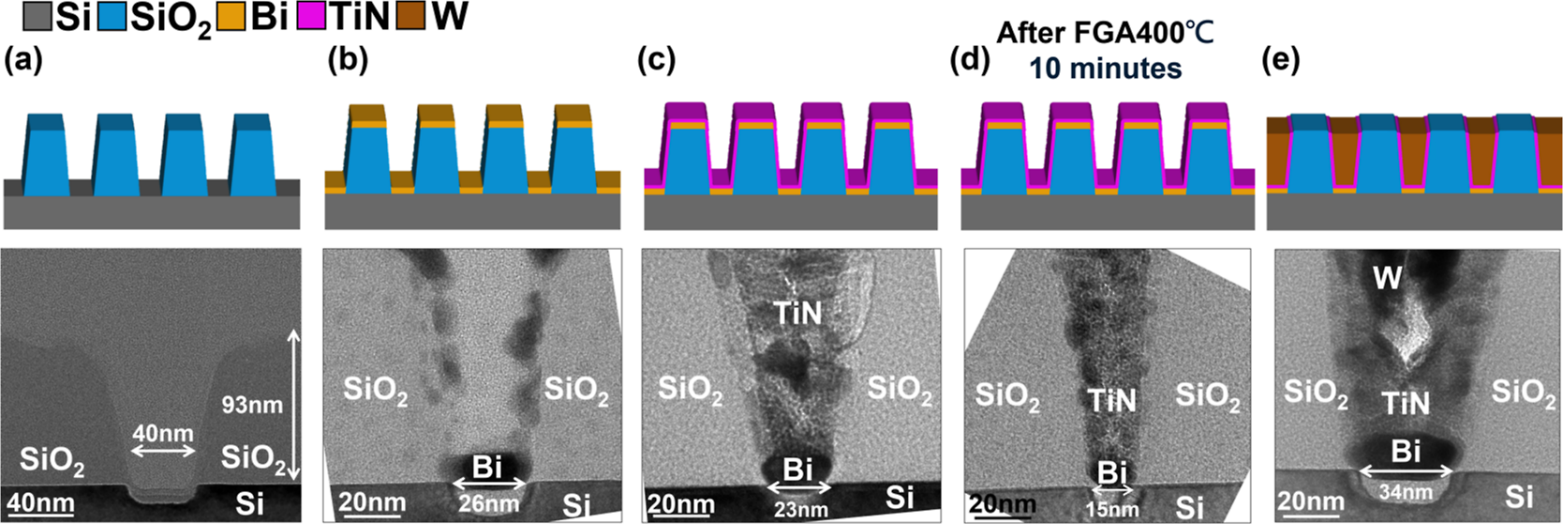
Integration of Bi with TiN barriers and W plugs for in contact holes. Top row includes the schematic representation, while the bottom row displays the TEM cross-section of the result. (a) ∼100 nm SiO_2_ was deposited on Si, patterned, and etched to form nanoscale trenches emulating contact holes. (b) Bi was deposited by PVD only at the bottom of the trench, as PVD is the highest throughput and most economical deposition method. As PVD results in nonconformal deposition, only minor deposition is observed on the contact walls. (c) Conformal ALD TiN encapsulates the Bi as a diffusion barrier. (d) Forming gas annealing at 400 °C for 10 min. Cross-sectional TEM confirms intact and fully confined Bi without evidence of outgassing or void formation. (e) W plug deposition by PVD fills the plug, mimicking standard contact metallization. Voids in the center of the W plug originate from nonconformal PVD fill and can be easily eliminated in a full process flow. Bi contacts integrated with standard TiN/W plugs are compatible with BEOL thermal budget as demonstrated in this integration test.

Figure 5 thus demonstrated that Bi contacts encapsulated by ALD TiN can withstand standard BEOL-like temperature and chemistry (400 °C, 10 min), confirming both material confinement and process compatibility. These findings bridge the gap between material-level stabilization (Figures 1 and 2) and device-level demonstrations (Figures 3 and 4), validating that low-*R*_C_ semimetal contacts can be scaled into realistic integration schemes. In addition to confirming Bi presence in the contact hole, it is equally critical to confirm that no diffusion into surrounding dielectrics, channels, or barrier interfaces occurs during processing. Further detailed TEM analysis and study of the different annealing environment are included in Figures S16–S18.

To assess the thermal stability of the contact structures, an extended forming-gas anneal with a duration of 1 h at 400 °C is applied. The process schematic is shown in Figure 6a. Cross-sectional TEM imaging after the extended annealing (Figure 6b) confirms that Bi remains structurally intact and confined within the SiO_2_ trench beneath the TiN barrier, with no evidence of void formation, delamination, or out-diffusion. Corresponding EDX elemental mapping (Figure 6c) includes a representation of Bi, Ti, and O in the same graph as well as their respective separate depictions (Figure 6d). Bi is confirmed to remain localized within the trench, indicating effective confinement under extended thermal stress. Uniform Ti and N signals confirm the conformality and continuity of the ALD TiN barrier, while O mapping reveals no interfacial oxidation at the Bi/TiN interface—ruling out oxidation-induced blocking and further affirming the barrier’s efficacy. The surrounding dielectric stack remains chemically and structurally intact, as well, to confirm the success of the integration strategy.

**Figure 6:**
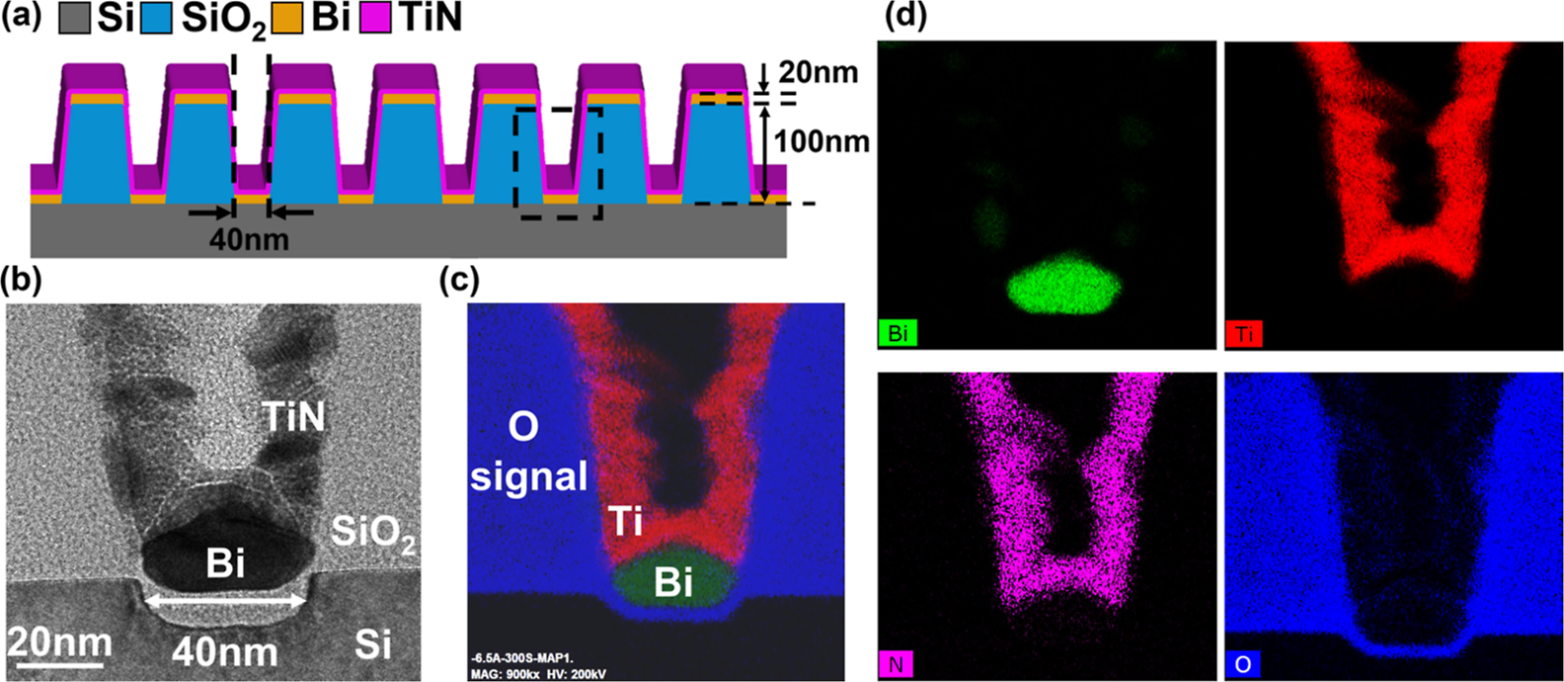
Extended annealing validates long-term robustness of Bi/TiN confined contacts. (a) Process schematic of the SiO_2_ trench, identical to Figure 5, used to evaluate stability under prolonged thermal annealing. (b) Cross-sectional TEM after 400 °C forming gas annealing for 1 h further confirms Bi contacts remain intact and fully confined within the contact hole under the ALD TiN barrier, without evidence of outgassing, voiding, or morphological degradation. (c) EDX elemental mapping of Bi/TiN metal plug structure after 1 h FGA (400 °C). Bi remains localized within the SiO_2_ trench beneath the TiN barrier, with no diffusion or oxidation detected, confirming excellent chemical stability under extended BEOL annealing. (d) EDX maps of Ti, Si, N, and O verify the uniformity of the TiN barrier and the integrity of the dielectric trench. Together, these results demonstrate that Bi contacts encapsulated by TiN barriers withstand extended BEOL annealing (400 °C, 1 h). Bi remains confined within the trench, while uniform Ti/N signals confirm a continuous ALD TiN barrier. The O map shows no oxidation at the Bi/TiN interface, indicating that Bi confinement arises from the barrier integrity rather than interfacial oxidation.

We have thus confirmed that Bi can be implemented as a first-layer metal in a contact scheme compatible with BEOL-like thermal budget and chemistry for transistors with a MoS_2_ monolayer channel. Although high selectivity etching methods for patterning oxide dielectrics while preserving 2D channels are still under development, the integration outlined here by using contact holes provides an experimental demonstration of a semimetal contact—Bi—being successfully confined by a diffusion barrier throughout extended BEOL annealing conditions. Further process optimization, including selective chemical–mechanical polishing (CMP) for Bi residue removal and contamination control during Bi metal deposition in a fabrication environment, remains an important subject for future work. In contrast to previous literature,^36,51–53^ which rarely addresses extended thermal stability under BEOL constraints, our results uniquely establish that Bi/TiN stacks offer both material robustness and process compatibility, advancing the feasibility of monolithic 3D integration schemes incorporating 2D semiconductors. Beyond thermal stability, a more quantitative assessment of contact reliability—such as direct electromigration lifetime and failure-threshold measurements—would be highly valuable. However, such evaluations require fully functional devices with an active channel (e.g., monolayer MoS_2_) capable of sustaining controlled current stress. At present, this is limited by the absence of a mature etch-stop process that can reliably terminate on the 2D channel beneath the dielectric without inducing damage. Addressing this integration challenge represents an important direction for future process development.

## Conclusion

In summary, we have demonstrated that confining Bi is a viable strategy for low *R*_C_ thermally stable contacts to monolayer MoS_2_ while applying BEOL temperature and chemical environment. While a lot of literature is focused on demonstrating that transistors can be fabricated with a low thermal budget to purportedly integrate them on top or within Cu interconnect layers, little has been done to find strategies to maintain device performance while integrating the subsequent interconnects on the transistors. The approach proposed here enables semimetal Bi contacts to simultaneously deliver ultralow *R*_C_ (<200 Ω·μm), high drive current, and resilience to BEOL thermal budgets. Through confinement of the Bi either through AlO_*x*_ capping or through TiN barriers, the contacts remain structurally and electrically intact after 400 °C forming-gas annealing. Transmission line method analysis confirms excellent contact resistance across tens of devices, both before and after exposure to BEOL-like conditions. Postannealing device operation further validates not only the thermal robustness of confined Bi contacts, but also the intrinsic stability of monolayer MoS_2_ channels—demonstrating that 2D transistors can endure BEOL stress without degradation.

While this work establishes a scalable pathway for BEOL integration of 2D TMD transistors—resolving the long-standing trade-off between low *R*_C_ and thermal stability—further progress in dielectric etch processes with high selectivity to 2D channels remains essential for full realization of 3D integration with true metal plug embedding in the device architectures.

## Methods

### Materials Growth

Monolayer MoS_2_ films were synthesized on *c*-plane sapphire substrates with a mis-cut angle using a three-zone chemical vapor deposition (CVD) system. Prior to growth, the substrates were annealed in an ambient atmosphere at 1100 °C for 6 h to form surface atomic step terraces. MoO_3_ powder (99.95%, Alfa Aesar) and sulfur powder (99.999%, Alfa Aesar) were used as the metal and chalcogen precursors, respectively. During the deposition process, the substrate temperature was maintained at 950 °C, while the MoO_3_ and sulfur source zones were heated to 680 and 160 °C, respectively. The growth was conducted for 10 min under a base pressure of 30 Torr using argon as the carrier gas (See Figure S19).

### Dry Transfer Process

Monolayer MoS_2_ single crystals grown on 2-in. Sapphire wafers were diced into 1 × 1 cm^2^ coupons. A PMMA A4 950 k layer was spin-coated (700 rpm for 10 s followed by 1600 rpm for 90 s, without soft baking). The photoresist at the coupon edges was removed, and thermal release tape (TRT) was attached to the surface. The sample was immersed in 1 M NaOH at 75 °C for 1 h to detach the MoS_2_ from sapphire. The detached film with TRT was rinsed in deionized (DI) water to remove residual NaOH, dried with N_2_, and stored in vacuum overnight to eliminate moisture. The MoS_2_/TRT stack was then laminated onto the target substrate using a PDMS stamp to enhance adhesion. The sample was baked at 120 °C to release the TRT, leaving MoS_2_ with the PMMA coating on the target. PMMA was removed by acetone at room temperature overnight, followed by IPA rinsing.

### Device Fabrication

Substrates with predefined pad patterns were cleaned by ultrasonication in acetone for 5 min, rinsed in IPA, and treated with O_2_ plasma (25 W, 1 min). After the dry transfer process, samples were annealed in forming gas (5% H_2_/95% N_2_) at 200 °C for 50 min (∼3.8 × 10^–3^ Torr) to remove PMMA residue. Optical microscopy confirmed complete coverage of MoS_2_ over the pads. The active regions were patterned using a Raith E-beam writer, developed in IPA/DI solution (50 s), and etched by reactive ion etching (O_2_/Cl_2_, 3 s). Raman and PL spectroscopy confirmed full removal of MoS_2_ outside the active regions. PMMA was stripped by hot acetone (80 °C, 2 h) followed by forming gas annealing (200 °C, 50 min).

Source/drain contacts were then defined by E-beam lithography, followed by deposition of 35 nm Bi using e-beam evaporation. Lift-off was carried out in hot acetone (85 °C) with ultrasonication if needed. Pristine devices were electrically characterized before AlO_*x*_ deposition. For capping, AlO_*x*_ was deposited by ALD under extended soak and purge conditions at low temperature to ensure conformal coverage without degrading device performance. Postdeposition, devices were annealed in a rapid thermal annealer (RTA) under forming gas or N_2_ at various temperatures and durations.

### Characterization

Electrical measurements were performed at room temperature (300 K) in vacuum (∼10^–2^ Torr) using a Lake Shore CPX-VF probe station and a Keysight B1500A Semiconductor Parameter Analyzer. Standard DC sweeps were used to extract transfer and output characteristics. Raman and PL spectra were collected with a Witec Alpha 300R system using a 532 nm excitation laser (spot size ∼1 μm). PL was measured near 670 nm with 5 mW excitation power and 3 s accumulation, while Raman spectra were measured at ∼400 cm^–1^ with 10 mW excitation power and 5 s accumulation.

### DFT Calculation

First-principles calculations are performed using the QuantumATK software package. The exchange–correlation interaction is treated within the generalized gradient approximation (GGA) using the Perdew–Burke–Ernzerhof (PBE) functional.^75^ A monolayer MoS_2_ is modeled under periodic boundary conditions, with a vacuum spacing of at least 20 Å applied normal to the layer to eliminate spurious interactions between periodic images. All atomic structures are fully relaxed until the residual forces on each atom fall below the convergence threshold. The relaxed structures are then used to extract intrinsic electronic properties of monolayer MoS_2_.

### Device Simulation

Device simulations^58^ incorporate material parameters extracted from first-principles calculations. Carrier transport is modeled with mobility degradation due to remote phonon and remote Coulomb scattering. Contact effects are captured by explicitly including the van der Waals gap at the metal–MoS_2_ interface, while carrier injection is described using a tunneling model based on the one-dimensional Schrödinger equation and the Wentzel–Kramers–Brillouin (WKB) approximation.^76^

### Contact Hole Structure Fabrication

Thermal oxidation was used to grow ∼100 nm SiO_2_ on an 8-in. Si wafer. Line trenches (width ∼50 nm, spacing ∼200 nm) were patterned by E-beam lithography and etched into the SiO_2_ using RIE with high selectivity to Si. Bi (∼15 nm) was deposited by e-beam evaporation to form contacts at the bottom of the trench. Due to the PVD process, minor Bi residues were observed on trench sidewalls. To emulate industrial integration, TiN barriers were deposited by plasma-enhanced ALD (200 °C, 150 W) to limit Bi diffusion. The structures were subjected to forming gas annealing to confirm confinement integrity. Finally, W plugs were deposited by PVD to fill the vias, followed by CMP planarization, mimicking the BEOL metallization flow.

## Supplementary Material



## Notes

Detailed structural and materials characterization of the bismuth confinement strategy, including area-dependent analysis, high-resolution TEM and EDS mapping under various annealing conditions, and cross-sectional studies before and after forming gas annealing. Additional fabrication process flow and Raman spectroscopy are provided. Statistical electrical characterization (threshold voltage, subthreshold swing, hysteresis, and transfer/output characteristics) before and after BEOL-compatible annealing is included, together with contact resistance analysis from TLM measurements, mobility extraction and benchmarking, and TCAD simulations of the Bi/MoS_2_ contact interface. Furthermore, wafer-scale MoS_2_ characterization by SHG and XRD, metal line resistance analysis, and extended thermal stress studies in 3D monolithic structures are provided (PDF)
