# Supplementary material for: Bismuth Confinement: A Strategy for Low Resistance and Good Thermal Endurance of Integrated Contacts to MoS2
Source: ACS Nano. 2026 Feb 24;20(12):9817–27. doi: 10.1021/acsnano.5c19217 (PMC13045353; doi:10.1021/acsnano.5c19217)
Supplement: Supplementary file 1 [file nn5c19217_si_001.pdf]

Supplementary Materials

Bismuth confinement: a strategy for low resistance and good thermal endurance of integrated contacts to MoS_2

Wen-Chia Wu[1,2], Terry Y.T. Hung[2], Fu-Kuo Hsueh[3], Bo-Heng Liu[4], Wei-Sheng Yun[2], Yun-Yan Chung[2], Yu-Ching Wang[1], Ze-Rui Lin[1], Meng-Zhan Li[2], Zih-Siang Jian[5], Szu-Huan Hsu[6,7], Jian-Chen Tsai[5], Jyun-Hong Chen[3], Chien-Wei Chen[4], Yiming Li[6,7], Wen-Hao Chang[5], Wei-Yen Woon[2], Chi-Chung Kei[4], Tuo-Hung Hou[3], Chao-Ching Cheng[2], Iuliana P. Radu[2], Chao-Hsin Chien[1*]*

[1]Institute of Electronics, National Yang Ming Chiao Tung University, Hsinchu 30010, Taiwan

[2]Corporate Research, Taiwan Semiconductor Manufacturing Company, Hsinchu 30075, Taiwan

[3]Taiwan Semiconductor Research Institute, Hsinchu 300091, Taiwan

[4] National Center for Instrumentation Research, National Institutes of Applied Research, Hsinchu 30076, Taiwan

[5]Department of Electrophysics, National Yang Ming Chiao Tung University, Hsinchu 30010, Taiwan

[6]Parallel and Scientific Computing Laboratory, National Yang Ming Chiao Tung University, Hsinchu 30010, Taiwan

[7]Institute of Communications Engineering, National Yang Ming Chiao Tung University, Hsinchu 30010, Taiwan

*To whom correspondence should be addressed.

E-mail: IRADU@tsmc.com, chchien@nycu.edu.tw

Supplementary Materials

1. **Area-dependent characterization of Bi confinement……………………………………………3**

2. **TEM/EDS analysis of Bi confinement at different annealing temperatures……………….…….5**

3. **EDS mapping of additional devices after FGA……………………….……………………….6**

4. **Device fabrication process flow & Raman spectrum………………………………….….……7**

5. **High-Resolution TEM Analysis of the Bismuth Confinement Device Structure…………..……….….…8**

6. **Cross-Sectional TEM of Bismuth on MoS_2 Before and After Forming Gas Annealing………..........9**

7. **Statistical electrical characterization before and after FGA — Subthreshold swing……………………10**

8. **Representative device transfer/output curves after FGA (I_D–V_G, I_D–V_D) ………….…….….11**

9. **Statistical Electrical Characterization before and after AlO_x Capping and Forming Gas Annealing.…12**

10. **Statistical electrical characterization — Threshold voltage.…………………………………14**

11. **Statistical electrical characterization — Hysteresis……………...……………………………15**

12. **Bismuth Metal Line Resistance Analysis and Layout Optimization……………………………….....16**

13. **Mobility extraction and benchmarking………………………………………………………......20**

14. **TCAD Simulation of Bi/MoS_2 Contact Interface before and after Forming Gas Annealing….…...….…21**

15. **Statistical Contact Resistance Analysis under BEOL-Compatible Annealing Conditions…….22**

16. **TEM/EDS line scan of pure Bi contacts in 3D monolithic structure……………………………24**

17. **TEM/EDS line scan of 3D monolithic structure under different annealing environments …...25**

18. **Step-by-step TEM analysis of 3D monolithic structure under extended annealing durations..26**

19. **Wafer-Scale Single-Crystal MoS_2 Characterization by SHG and XRD……………………...27**

20. **Reference…………………………………………………………….……………………...28**

Supplementary Materials

1. Area-dependent characterization of Bi confinement

(a)

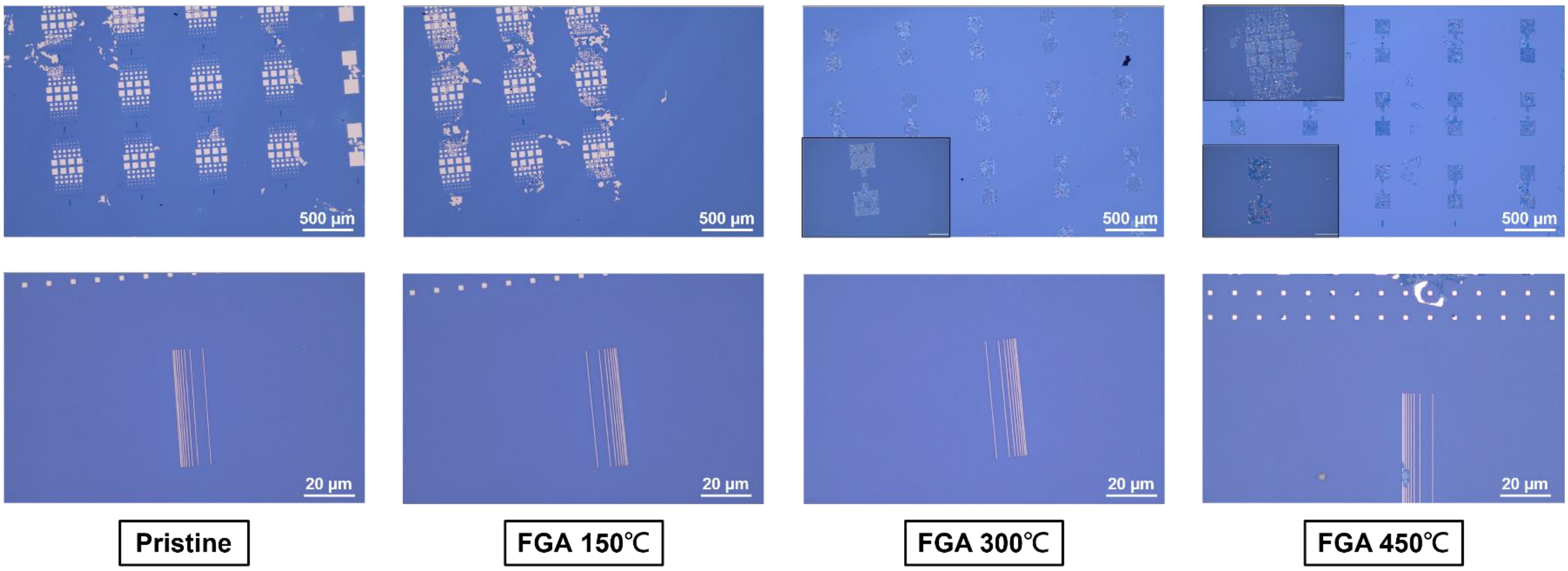

(b)

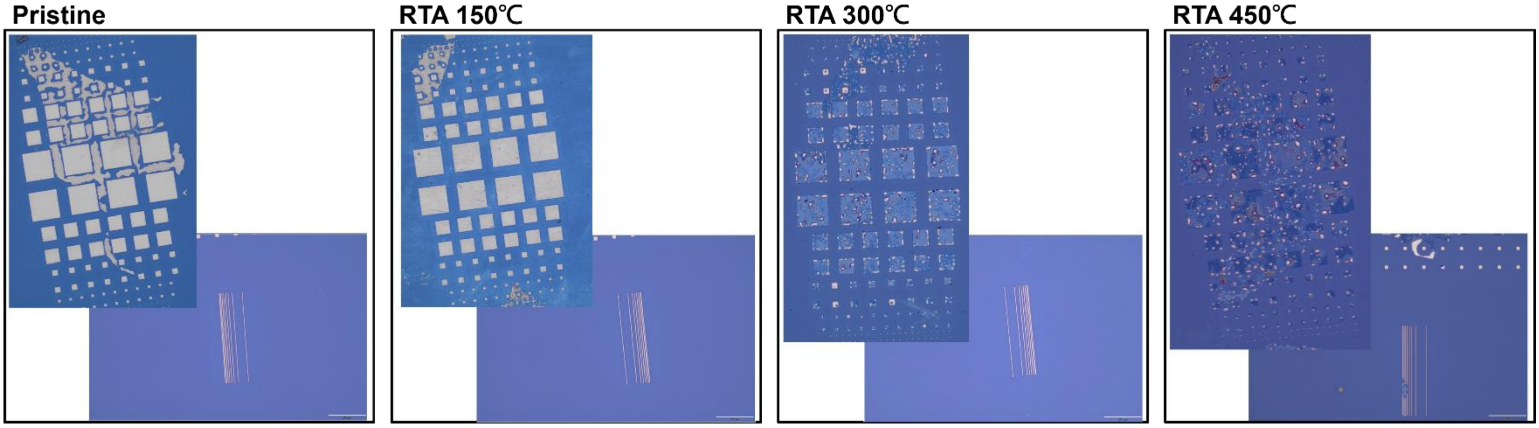

Area (um*um)	Total (# of device)	Pristine		RTA150℃		RTA300℃		RTA450℃	
		Alive (# of device)	Alive (%)	Alive (# of device)	Alive (%)	Alive (# of device)	Alive (%)	Alive (# of device)	Alive (%)
metal line 300nm	48	48	100.00%	48	100.00%	48	100.00%	48	100.00%
2*2 μm^2	240	240	100.00%	240	100.00%	226	94.17%	162	67.50%
5*5 μm^2	132	132	100.00%	132	100.00%	8	6.06%	0	0.00%
10*10 μm^2	96	96	100.00%	96	100.00%	10	10.42%	0	0.00%
25*25 μm^2	72	72	100.00%	72	100.00%	0	0.00%	0	0.00%
50*50 μm^2	24	24	100.00%	24	100.00%	0	0.00%	0	0.00%

Supplementary Materials

(c)

Well-confined

Area (μm^2)	metal line 600nm*48μm	2*2	5*5	10*10	25*25	50*50
Total device (#)	288	1140	792	576	432	144
Pristine	100%	100%	100%	100%	100%	100%
RTA150℃	100%	100%	100%	100%	100%	100%
RTA300℃	100%	94%	6%	10%	0%	0%
RTA450℃	100%	68%	0%	0%	0%	0%

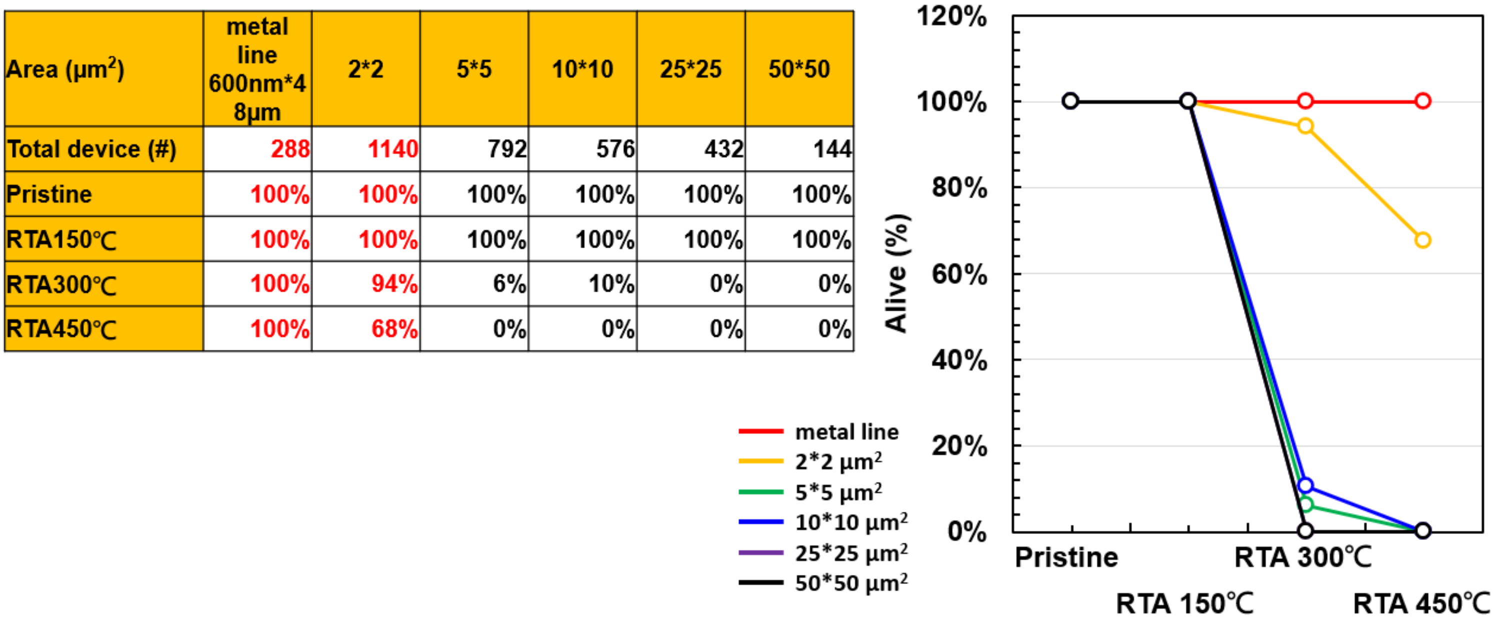

Figure S1. Optical analysis of area-dependent Bi confinement. (a) Optical images of Bi pads with varying lateral dimensions subjected to stepwise FGA from pristine to 450°C. (b) Representative optical images of Bi pads of different sizes with corresponding statistical analysis of survival fractions at different annealing temperatures. (c) Statistical summary of survival ratio as a function of pad size and annealing temperature, revealing a strong area dependence and indicating that pads of ~2 μm × 2 μm or smaller provide the most reliable confinement.

Supplementary Materials

2. TEM/EDS analysis of Bi confinement at different annealing temperatures

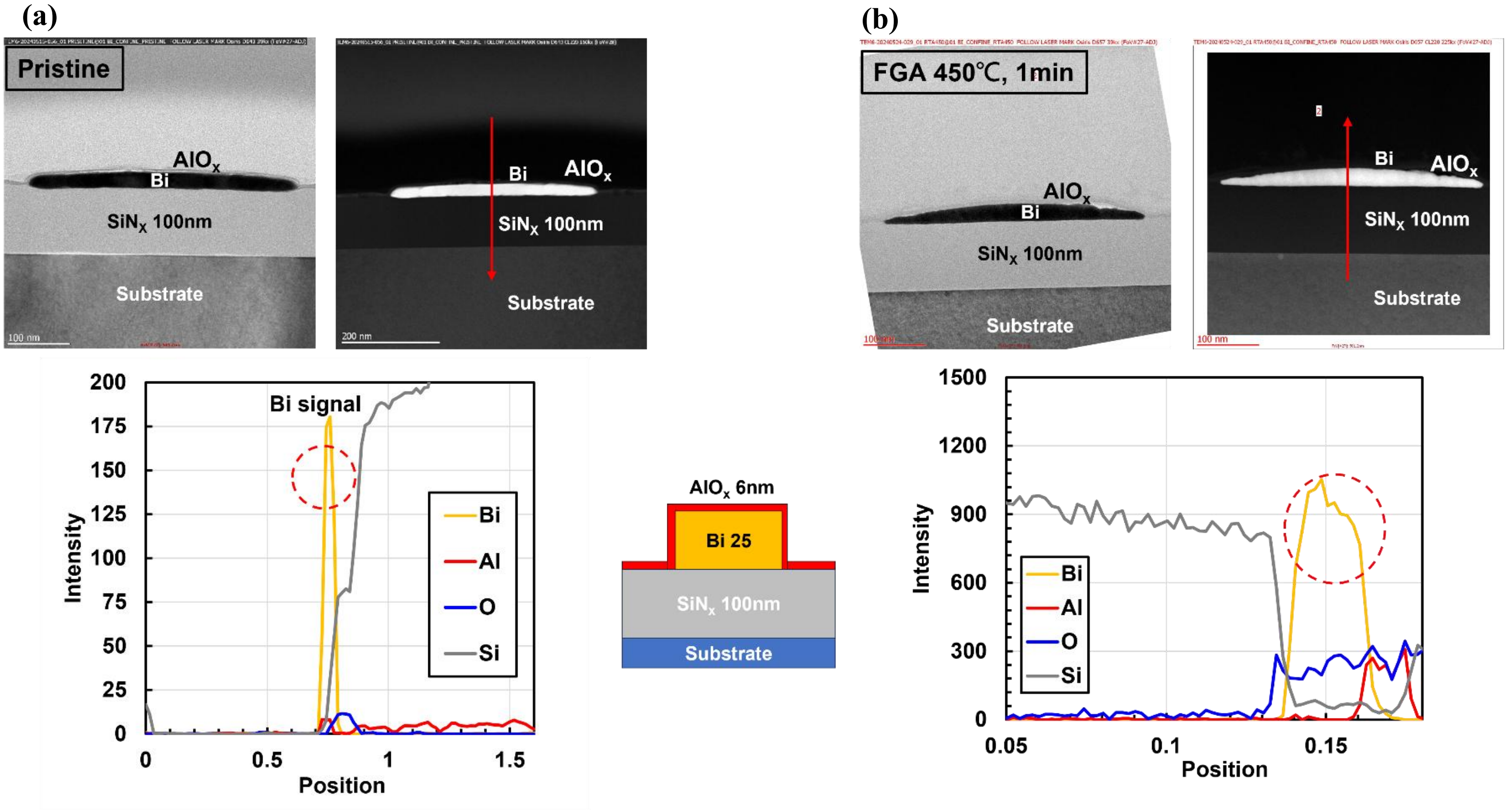

Figure S2. TEM and EDS line analysis of Bi confinement before and after FGA at 450°C. (a) TEM image and corresponding EDS line scan of a Bi metal line confined on SiN_x substrate with low-temperature ALD AlO_x capping, showing a strong Bi signal in the as-deposited state. (b) TEM image and EDS line scan of the same structure after FGA at 450°C, confirming that Bi remains well confined with a strong EDS signal.

Supplementary Materials

3. EDS mapping of additional devices after FGA

(a) **(b)**

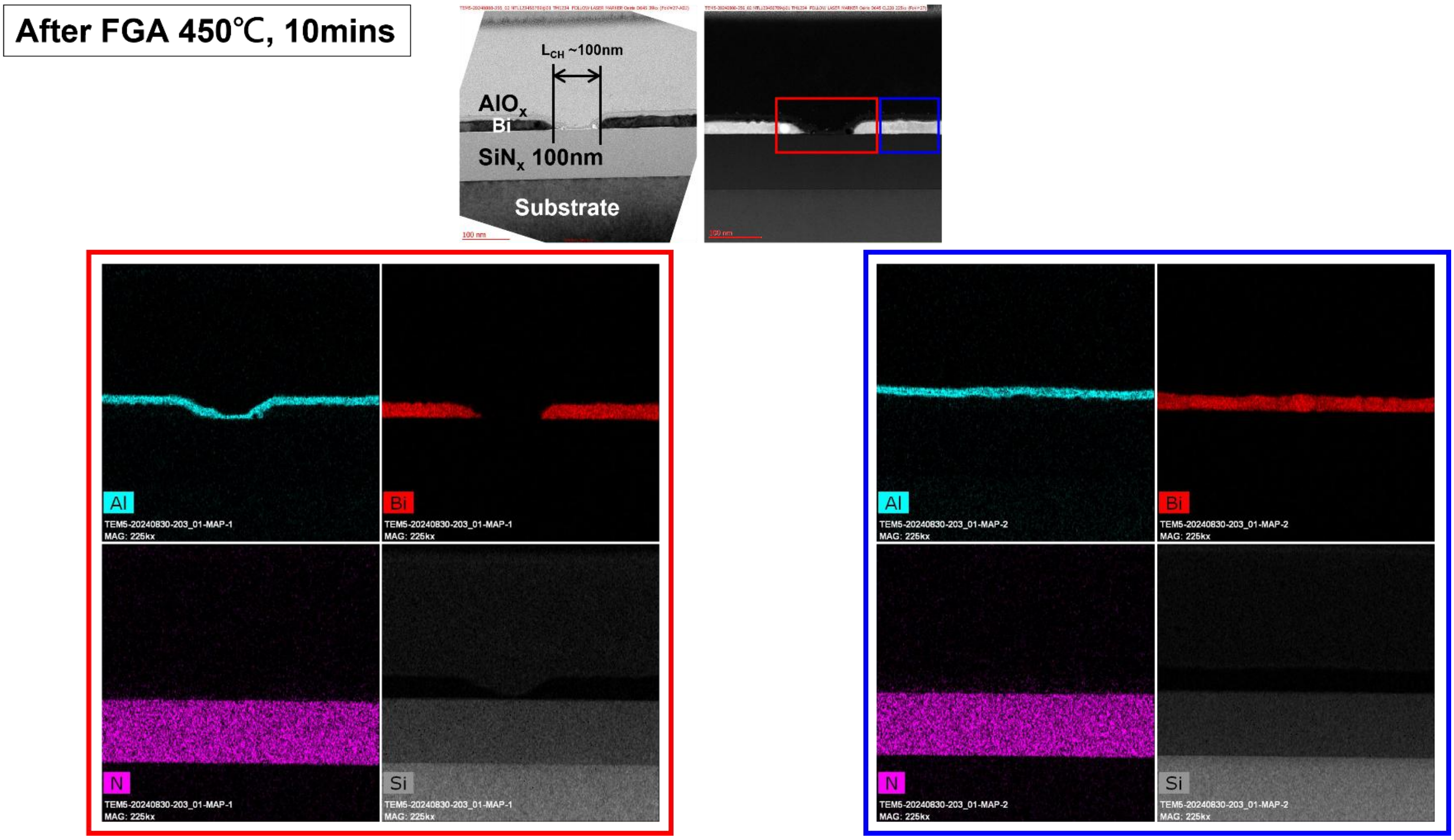

Figure S3. TEM/EDS mapping of Bi-confinement device on SiN_x substrate after FGA at 450°C for 10 min. (a) Channel region elemental mapping (Al, Bi, N, and Si), showing that Bi remains well localized in the contact region after annealing. (b) Source/drain metal-line region mapping with the same elemental signals, confirming that Bi confinement is maintained in the metal lines following FGA treatment.

Supplementary Materials

4. Device fabrication process flow & Raman spectrum

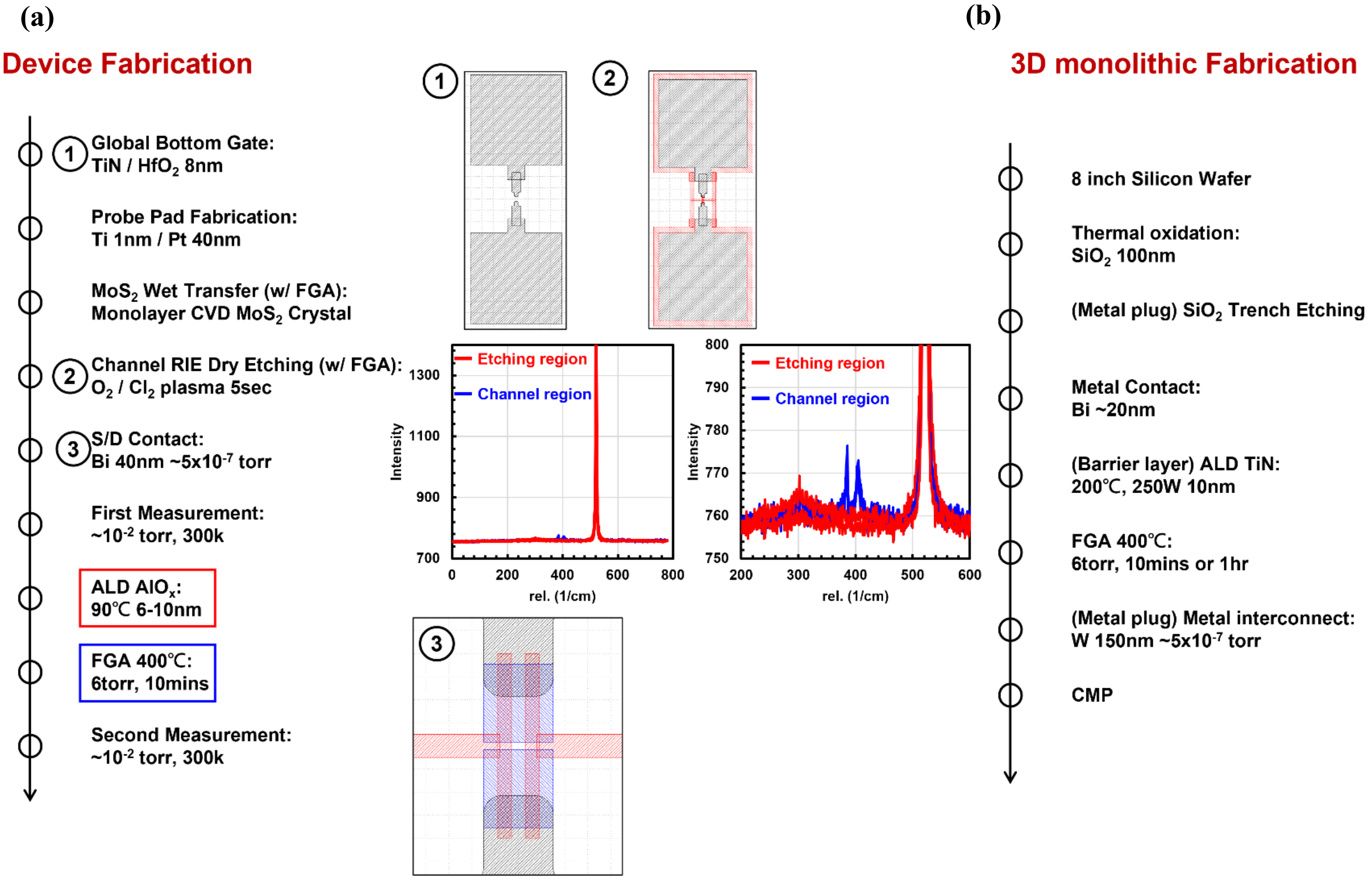

Figure S4. Device fabrication process flow. (a) CVD-grown monolayer MoS_2 FETs before and after FGA treatment, along with Raman spectra recorded before and after channel region definition. (b) Process schematic of the 3D monolithic structure used for Bi confinement studies.

Supplementary Materials

5. High-Resolution TEM Analysis of the Bismuth Confinement Device Structure

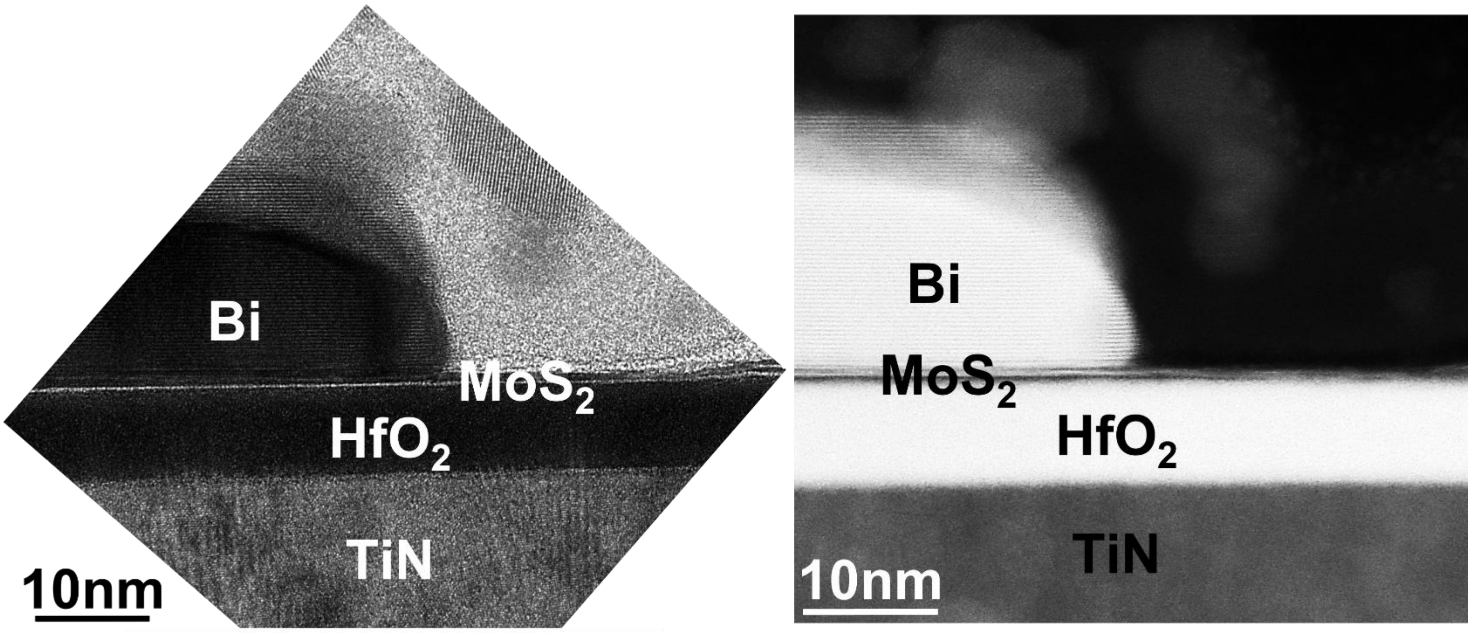

Figure S5. High-resolution TEM analysis of the Bismuth confinement structure. High-resolution cross-sectional transmission electron microscopy (TEM) and high-angle annular dark-field scanning TEM (HAADF-STEM) images taken from one side of the contact region of the Bismuth-confinement device shown in Fig. 3b. No observable metal diffusion or interfacial degradation is detected at the Bi/MoS_2 or Bi/AlOx, supporting the structural integrity and thermal stability of the confinement strategy.

Supplementary Materials

6. Cross-Sectional TEM of Bismuth on MoS_2 Before and After Forming Gas Annealing

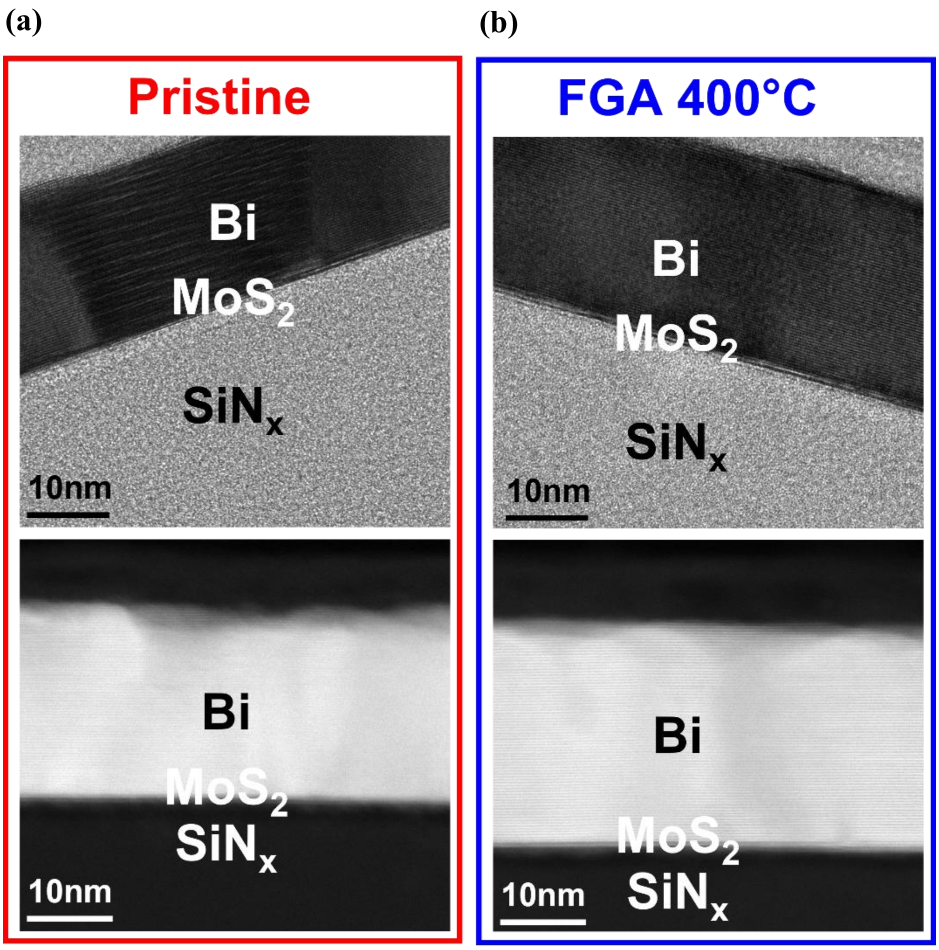

Figure S6. Cross-sectional TEM analysis of Bismuth confinement before and after forming gas annealing (FGA). (a) Cross-section transmission electron microscopy (TEM) images of the Bismuth (Bi) contact on monolayer MoS_2 in the pristine state, including conventional TEM (top) and Z-contrast (bottom) imaging. The Bi metal forms a continuous and well-defined interface with the underlying monolayer MoS_2 and SiN_x dielectric. (b) Corresponding TEM images after forming gas annealing (FGA) at 400 °C, showing that the Bi metal layer remains continuous and crystalline, with no observable diffusion, agglomeration, or interfacial degradation. These results indicate that the confined Bi contact preserves its structural integrity on monolayer MoS_2 under BEOL-compatible thermal processing.

7. Statistical electrical characterization before and after FGA — Subthreshold swing

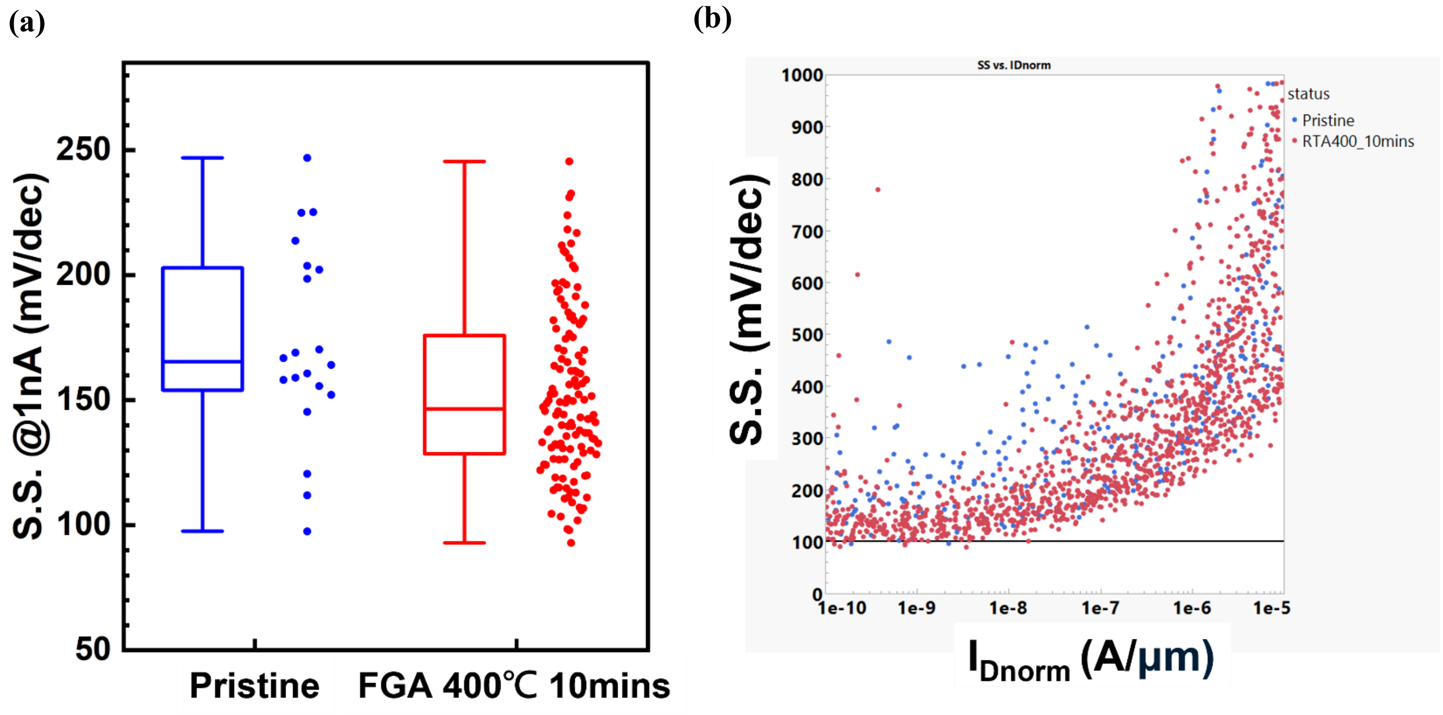

Figure S7. Statistical analysis of subthreshold swing before and after FGA at 400°C for 10 min. (a) S.S. @1nA for channel lengths ranging from 100 nm to 1 μm, showing no degradation after FGA, which indicates negligible interface degradation. (b) S.S. plotted against drain current (I_D) on a logarithmic scale, demonstrating that the minimum S.S. in HfO_2 with EOT ≈ 2 nm is ~100 mV/dec and remains unchanged after FGA treatment.

Supplementary Materials

8. Representative device transfer/output curves after FGA (I_D–V_G, I_D–V_D)

(a) (b)

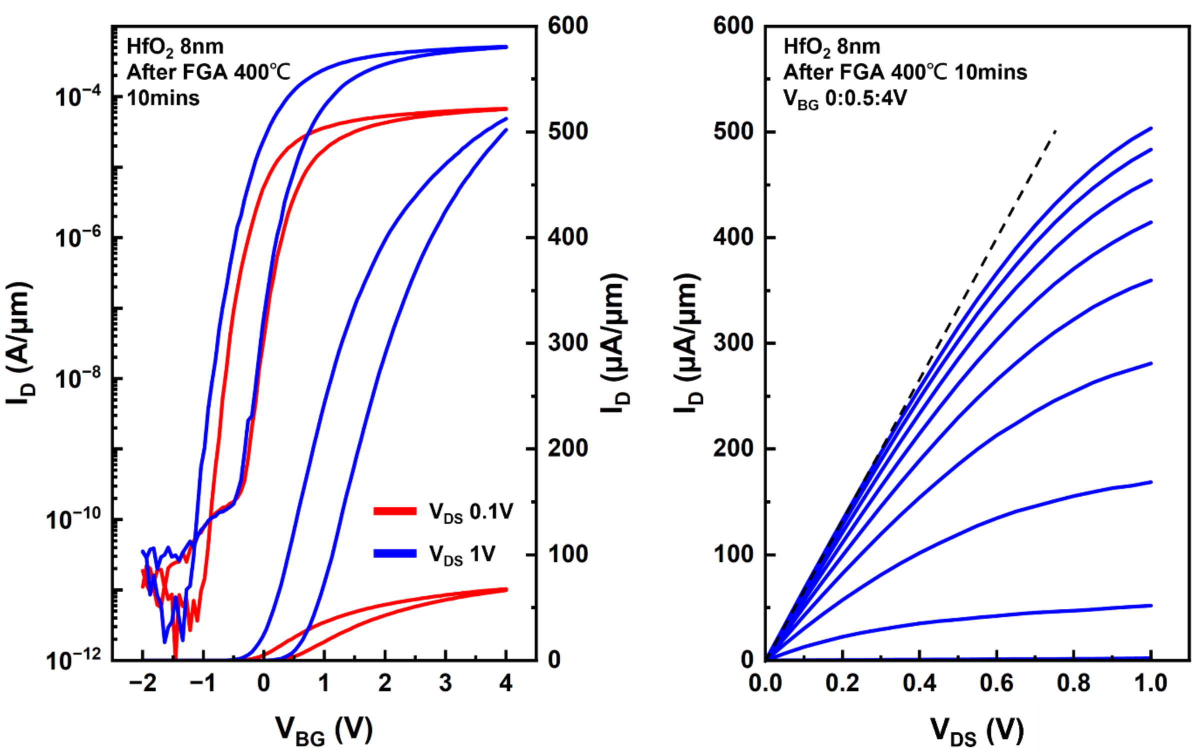

Figure S8. Representative device characterization before and after FGA at 400°C for 10 min. (a) Transfer characteristics measured at V_{DS} = 0.1 V (red) and 1 V (blue), showing high performance with negligible V_{TH} shift. Hysteresis of ~0.5–0.8 V persists in both cases, therefore DIBL is not further discussed. (b) Output characteristics after FGA annealing retain ohmic injection with linear I_D–V_D behavior at low V_{DS}. Saturation is less pronounced due to residual Bi metal resistance, but overall device performance is preserved.

Supplementary Materials

9. Statistical Electrical Characterization before and after AlO_x Capping and Forming Gas Annealing

(a)

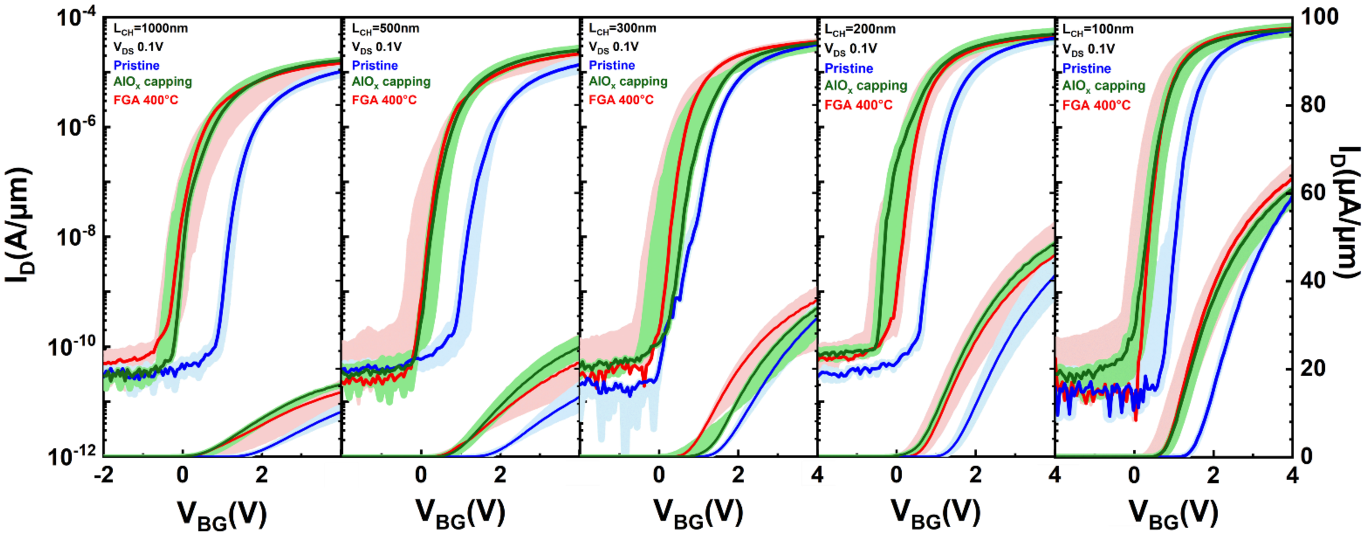

(b)

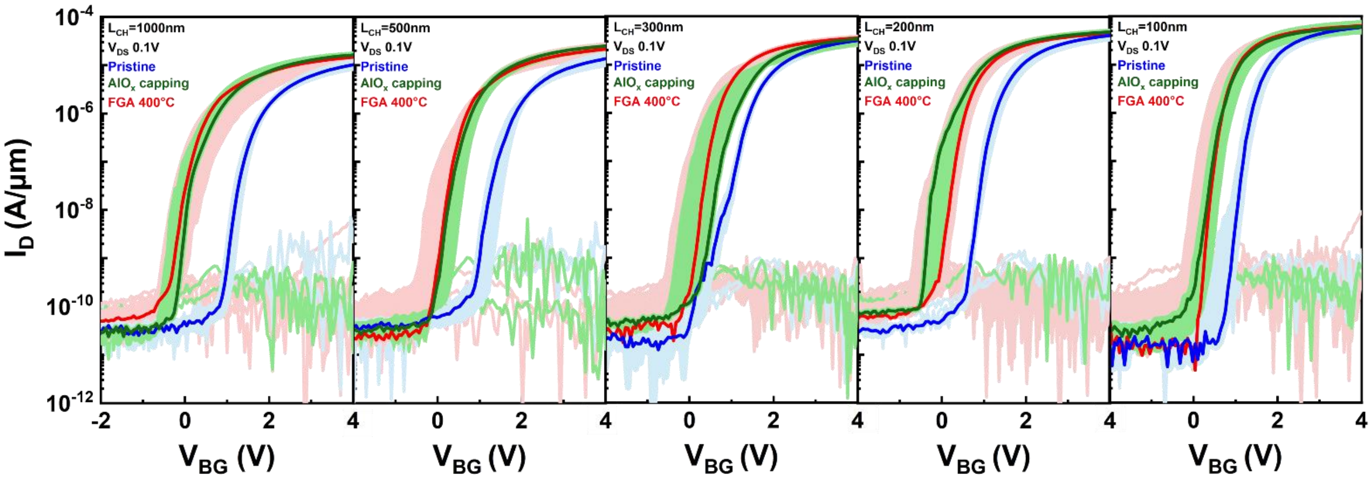

(c)

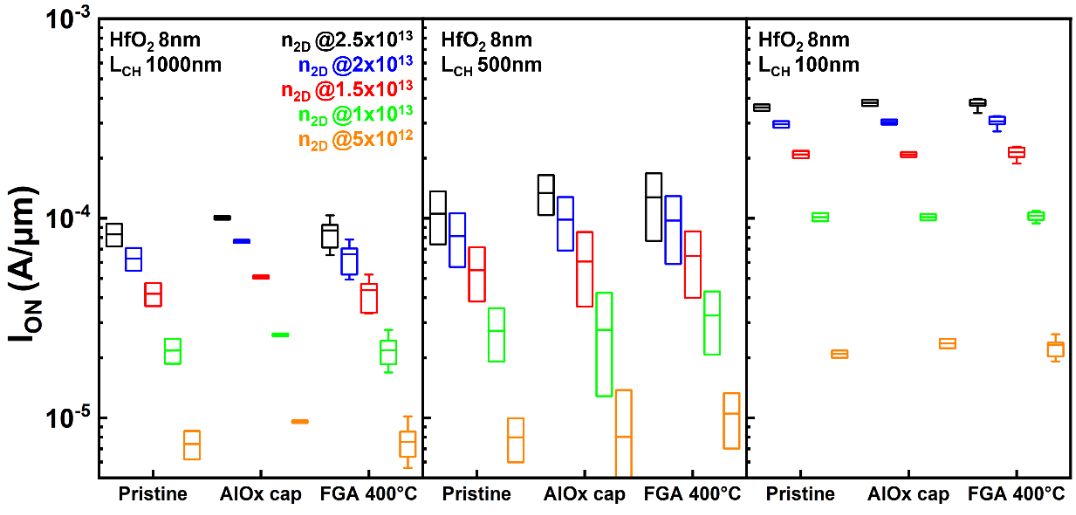

Supplementary Materials

(d)

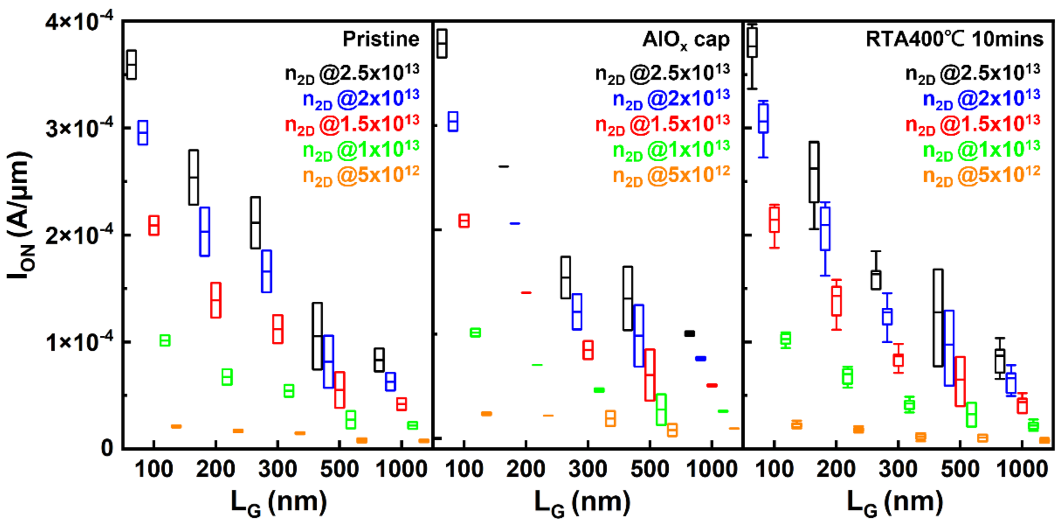

Figure S9. Statistical comparison of device characteristics before and after AlO_x capping and forming gas annealing (FGA). (a) Representative transfer characteristics (logarithmic and linear scales) measured at V_{DS}= 0.1 V for devices with different channel lengths (L_{CH}= 1000, 500, 300, 200, and 100 nm), including pristine devices (blue), devices with AlO_x capping (green), and devices after AlO_x capping followed by FGA at 400 °C (red). (b) Corresponding transfer characteristics in logarithmic scale together with gate leakage current, confirming that the observed performance variations are not correlated with increased gate leakage after AlO_x capping or FGA treatment. (c) Statistical comparison of on-current (I_{ON}) for representative channel lengths (L_{CH} = 1 μm, 500 nm, and 100 nm) extracted at carrier densities ranging from 2.5 × 10^{13} to 5 × 10^{12} cm^{-2} under pristine, AlO_x-capped, and FGA-treated conditions. (d) I_{ON} distribution as a function of channel length for all measured devices under the three processing conditions, demonstrating consistent current enhancement and limited device-to-device variation after AlO_x capping and subsequent FGA.

Supplementary Materials

10. Statistical electrical characterization — Threshold voltage

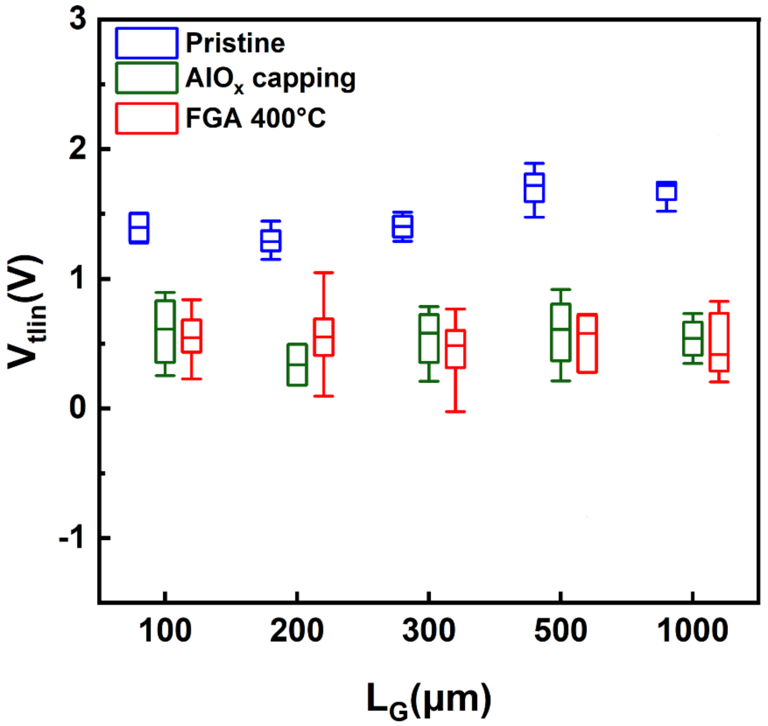

Figure S10. Statistical analysis of threshold voltage variation before and after AlO_x deposition and forming gas annealing (FGA). Statistical distribution of threshold voltage (V_{TH}) as a function of gate length (L_G) for pristine devices (blue), devices with AlO_x capping (green), and devices after AlO_x capping followed by FGA at 400 °C (red). A systematic negative shift in V_{TH} is observed after AlO_x deposition across all gate lengths, which is consistent with the presence of positive fixed charges[1,2] in the low-temperature ALD AlO_x capping layer, leading to additional electron accumulation in the MoS_2 channel. After FGA treatment, the V_{TH} distribution remains stable with comparable statistical variation.

11. Statistical electrical characterization — Hysteresis

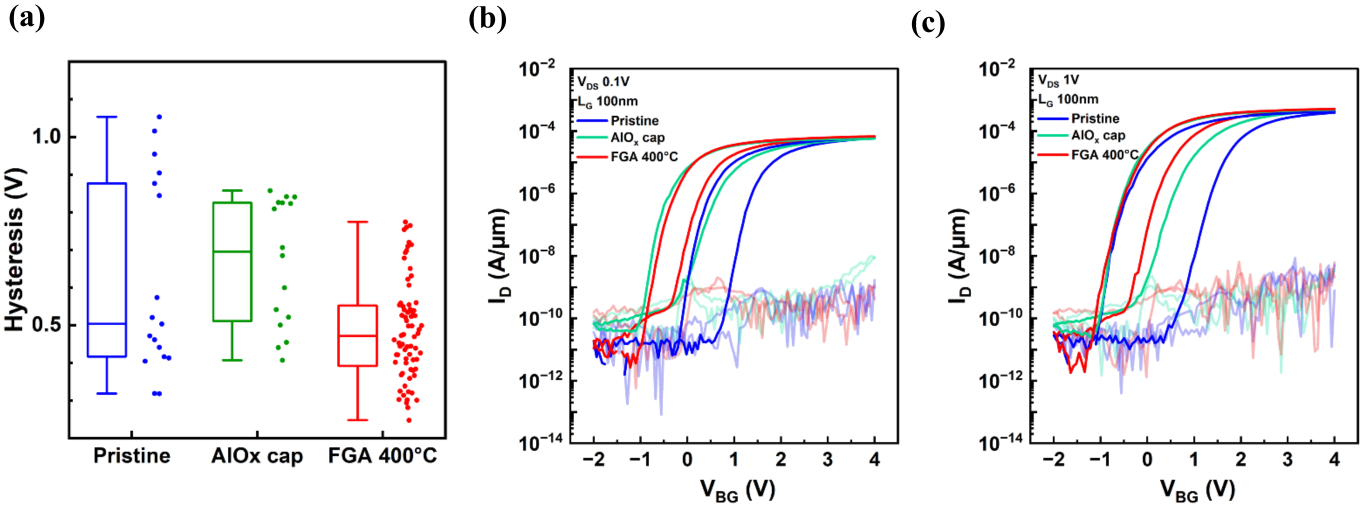

Figure S11. Statistical analysis of hysteresis before and after AlO_x deposition and forming gas annealing (FGA). (a) Statistical distribution of hysteresis extracted from forward and reverse transfer characteristics for pristine devices (blue), devices with AlO_x capping (green), and devices after AlO_x capping followed by FGA at 400 °C (red). The AlO_x-capped devices exhibit a comparable level of hysteresis to pristine devices, which is commonly observed in low-temperature ALD dielectrics due to poor dielectric quality. After FGA treatment, a reduced hysteresis with narrower statistical distribution is observed, indicating improved interfacial and dielectric stability[3–6]. (b,c) Representative transfer characteristics measured on the same device at V_{DS} = 0.1 V and 1 V, respectively, illustrating the evolution of hysteresis under pristine (blue), AlO_x-capped (green), and FGA-treated (red) conditions. The consistent hysteresis reduction after FGA across different drain biases further supports the role of post-annealing treatment.

Supplementary Materials

12. Bismuth Metal Line Resistance Analysis and Layout Optimization

(a)

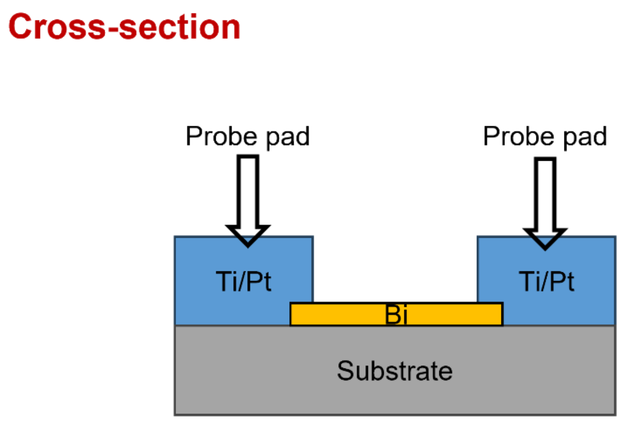

(b)

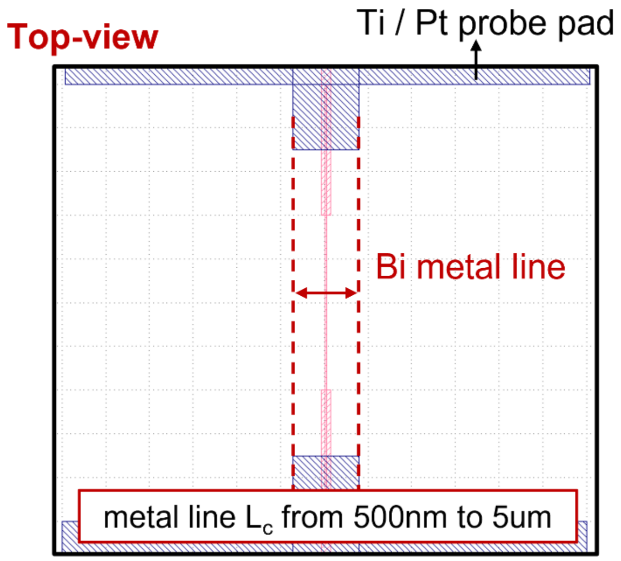

(c)

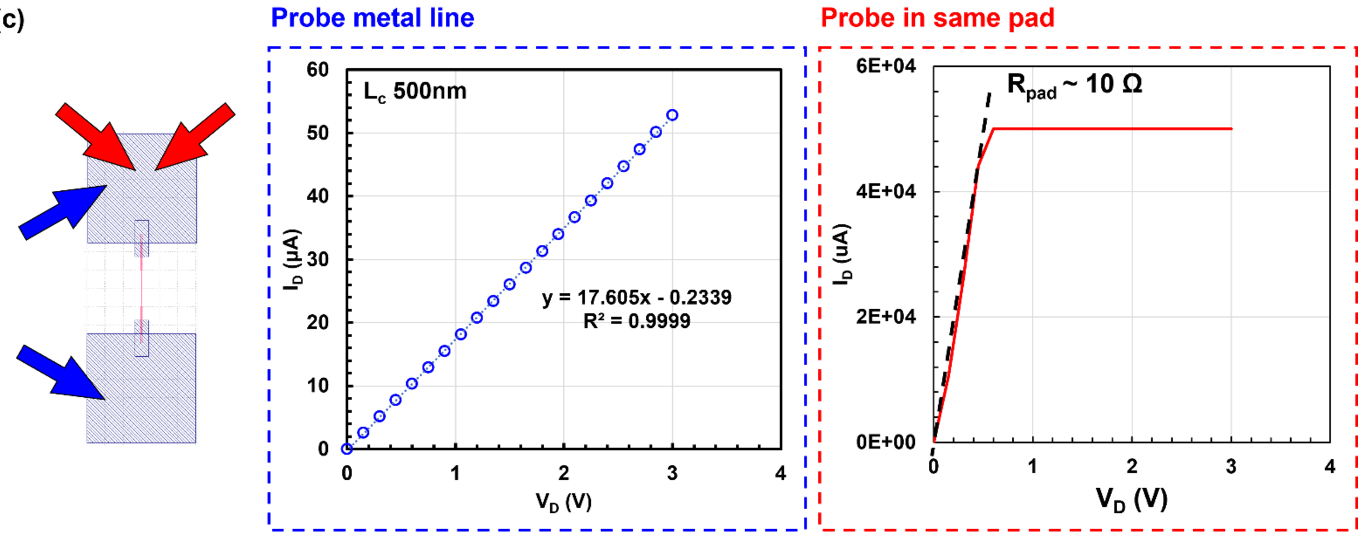

(d)

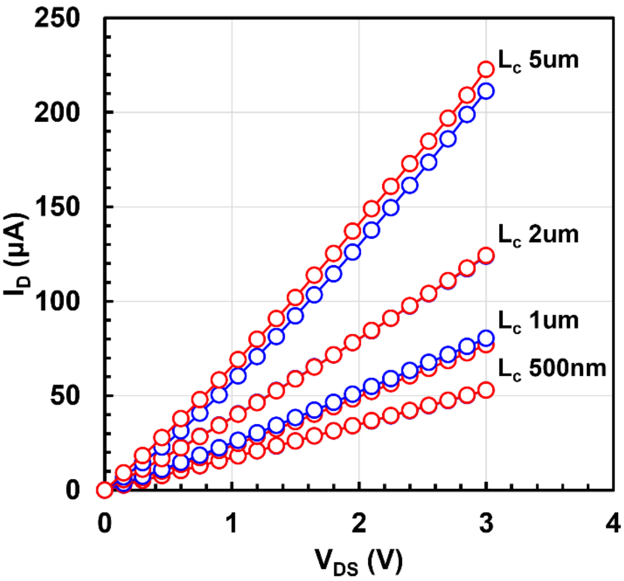

(e)

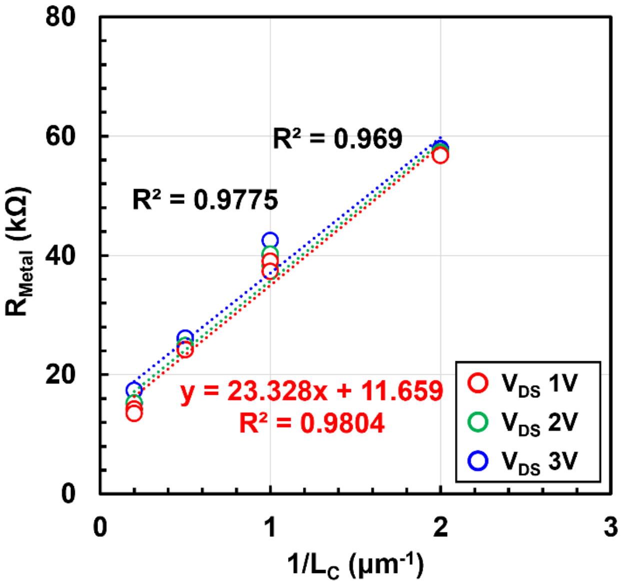

Supplementary Materials

Calculate the resistivity of Bi metal

(f)

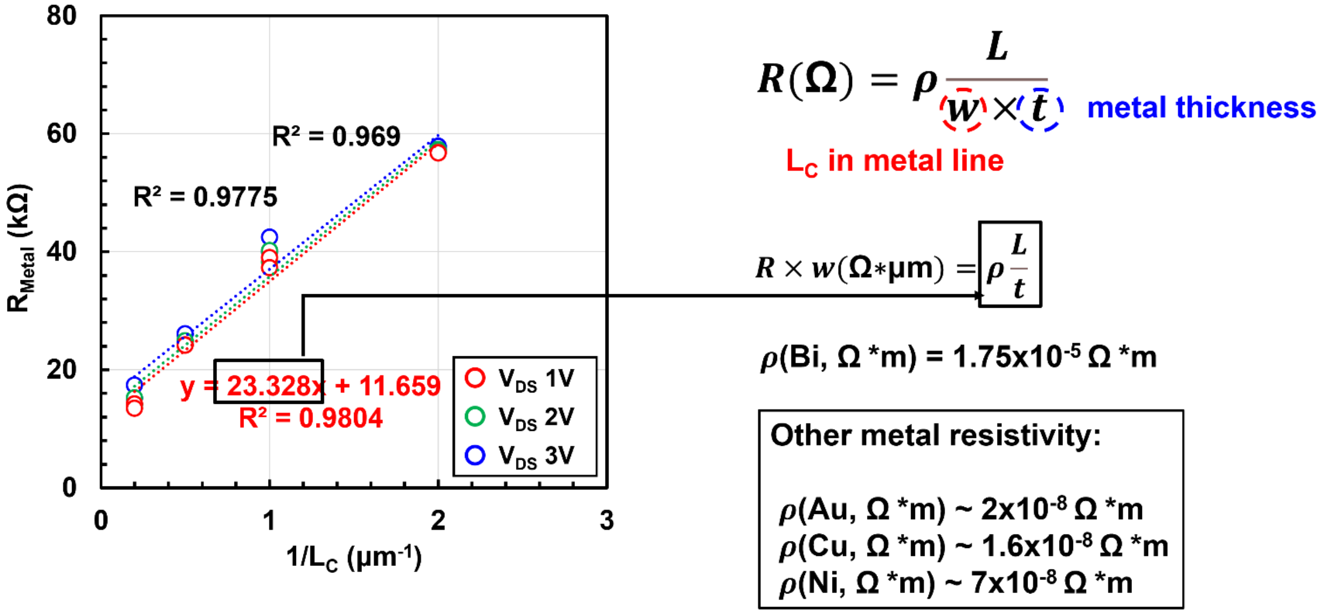

$$R(\Omega) = \rho \frac{L}{w \times t}$$

metal thickness

L_C in metal line

$$R \times w(\Omega * \mu m) = \rho \frac{L}{t}$$

ρ(Bi, Ω*m) = 1.75x10^{-5} Ω*m

Other metal resistivity:

ρ(Au, Ω*m) ~ 2x10^{-8} Ω*m
ρ(Cu, Ω*m) ~ 1.6x10^{-8} Ω*m
ρ(Ni, Ω*m) ~ 7x10^{-8} Ω*m

(g)

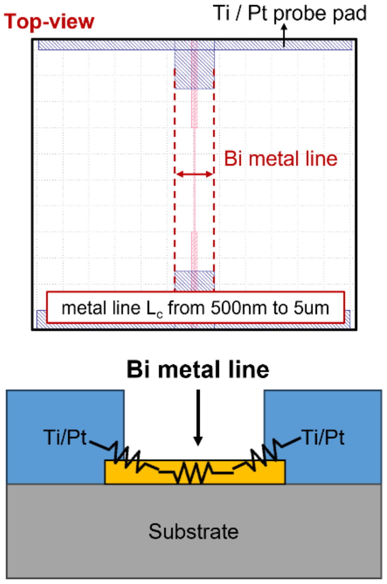

$R_{metal\ line} = 2*R_{interface} + R_{Bi,\ metal}$

Layout red area

Ex.1:
$R_{metal\ line}$ in L_c 500nm = 57 kΩ
$R_{Bi,\ metal}$ (red part) = 54.5 kΩ
=> $2R_{interface}$ = 2.5 kΩ

95% resistance
come from Bi metal line
(Reduce Bi metal line resistance)

Same $R_{interface}$

Ex.2:
$R_{metal\ line}$ in L_c 5um = 15 kΩ
$R_{Bi,\ metal}$ (red part) = 12.5 kΩ
=> $2R_{interface}$ = 2.5 kΩ

Supplementary Materials

Optimized layout design

(h)

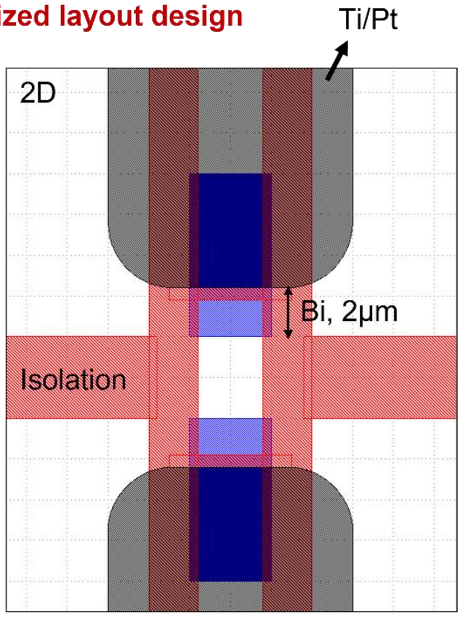

Statistical metal line before and after FGA

(i)

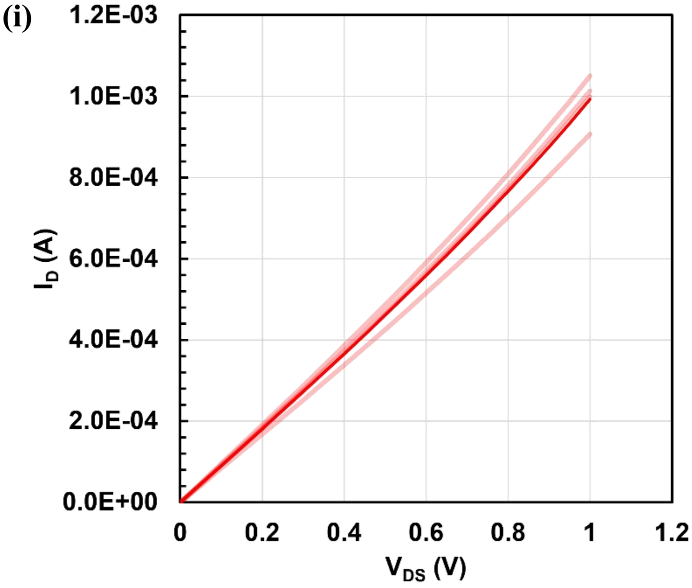

Figure S12. Bismuth metal line resistance analysis and layout optimization. (a) Cross-sectional schematic of the metal line test structure, where the metal line is formed by Bismuth (Bi) and the probe pads are composed of Ti/Pt. (b) Top-view schematic of the metal line device layout, showing Bi metal lines with different line widths (L_C) ranging from 500 nm to 5 µm. (c) Representative output characteristics used to decouple the resistance contributions from the probe pads and the Bi metal line. Linear I–V behavior measured within the same probe pad confirms negligible probe pad resistance (~10 Ω), whereas the dominant resistance originates from the Bi metal line. (d) Output characteristics of Bi metal line devices with different L_C, indicating a clear metal-line-width-dependent resistance behavior. (e) Extracted metal line resistance (R_{metal}) as a function of inverse metal line width ($1/L_C$) under different drain biases (V_{DS} = 1, 2, and 3 V), showing good linearity and confirming ohmic transport in the Bi metal line. (f) Metal resistance model based on $R = \rho \cdot L/(w \cdot t)$, where ρ is the metal resistivity, L is the metal line length, w is the line width, and t is the metal thickness. The extracted resistivity of Bi (~1.7×10^{-5} Ω·m) is significantly higher than that of

commonly used metals such as Au, Cu, and Ni. (g) Resistance decomposition analysis illustrating that the total measured resistance consists of the Bi metal line resistance and the interface resistance between the Ti/Pt probe pad and the Bi metal. After accounting for the intrinsic Bi resistivity, the resistance is found to be predominantly governed by the Bi metal line, while the interface resistance remains nearly constant across different L_C. (h) Optimized device layout design based on the metal resistance analysis, where the effective Bi metal line length and width are minimized to reduce series resistance. (i) Statistical output characteristics of the optimized Bi metal line devices, demonstrating stable and reduced metal line resistance on the order of ~1 kΩ after layout optimization.

13. Mobility extraction and benchmarking

$$R_{\text{tot}} = 2R_{\text{c}} + \rho\frac{L}{w},\ \rho = (nq\mu)^{-1}. \qquad (1)$$

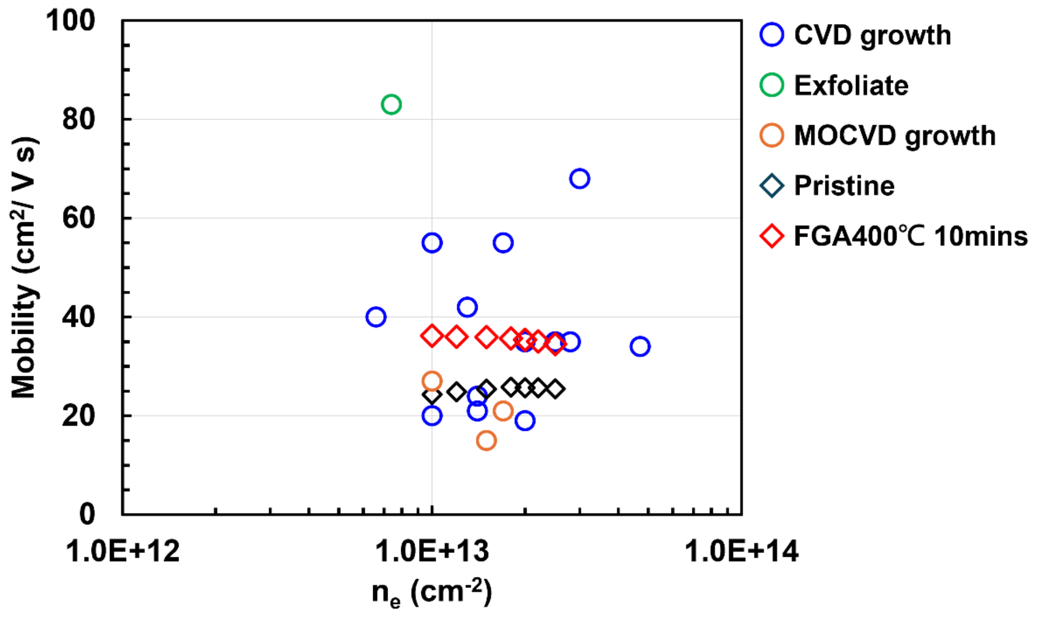

Figure S13. TLM mobility extraction and benchmarking. Equation (1) illustrates the transfer length method (TLM) used to decouple channel resistance and contact resistance from the total device resistance. From the slope of R_{TOT} versus L_{CH}, the sheet resistivity and intrinsic carrier mobility can be extracted without R_C interference. In Benchmark of intrinsic mobility for our devices (~27 cm²/V·s pristine, increasing to ~32 cm²/V·s after FGA at 400 °C for 10 min) compared to reported values from CVD[1,7–19], MOCVD[17,20,21], and exfoliation[22–24] methods. The post-anneal mobility enhancement is attributed to partial defect passivation or reduced carrier scattering[25–28].

TSMC Property

14. TCAD Simulation of Bi/MoS_2 Contact Interface before and after Forming Gas Annealing

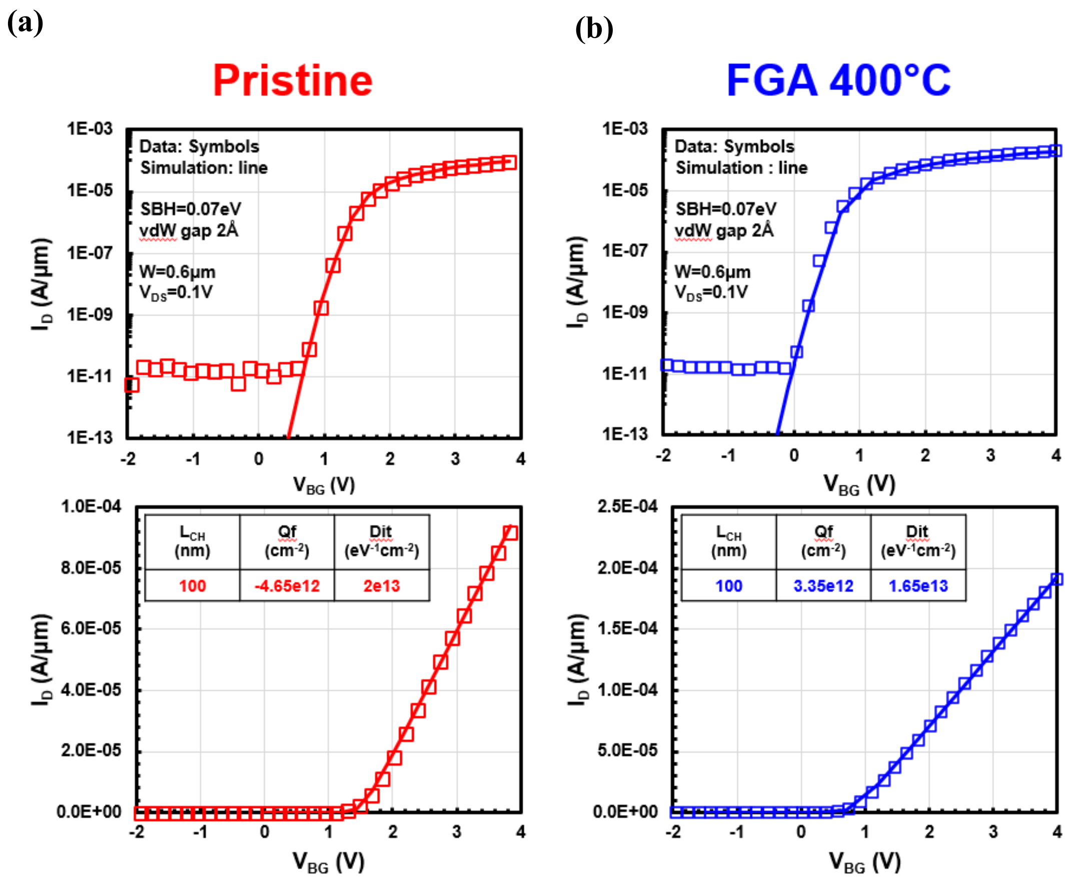

Figure S14. TCAD simulation of short-channel devices without metal line resistance before and after forming gas annealing (FGA). (a) TCAD-simulated transfer characteristics of the shortest-channel-length device with Bi contacts before FGA treatment, showing good agreement with experimental data. The simulation is performed using a Schottky barrier height (SBH) of 70 meV and a van der Waals (vdW) gap of 2 Å at the Bi/MoS_2 interface. (b) Corresponding TCAD simulation after FGA treatment at 400 °C using the same SBH (70 meV) and vdW gap (2 Å). The simulated transfer characteristics remain consistent with experimental results. The comparable fitting parameters before and after FGA indicate that the Bi/MoS_2 contact interface preserves a clean and stable van der Waals gap, and that the FGA process does not introduce noticeable modification to the Schottky barrier height or interfacial separation.

Supplementary Materials

15. Statistical Contact Resistance Analysis under BEOL-Compatible Annealing Conditions

(a)

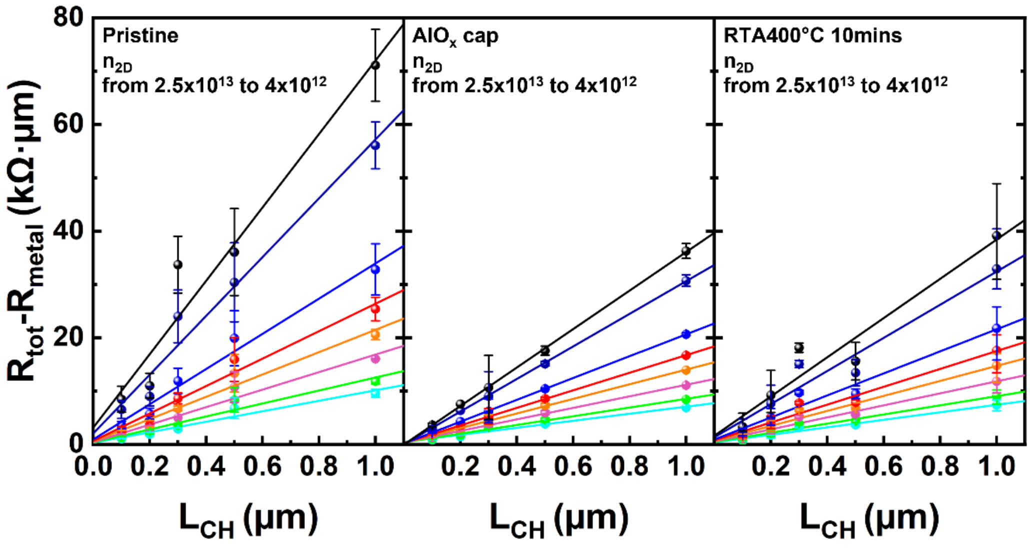

(b)

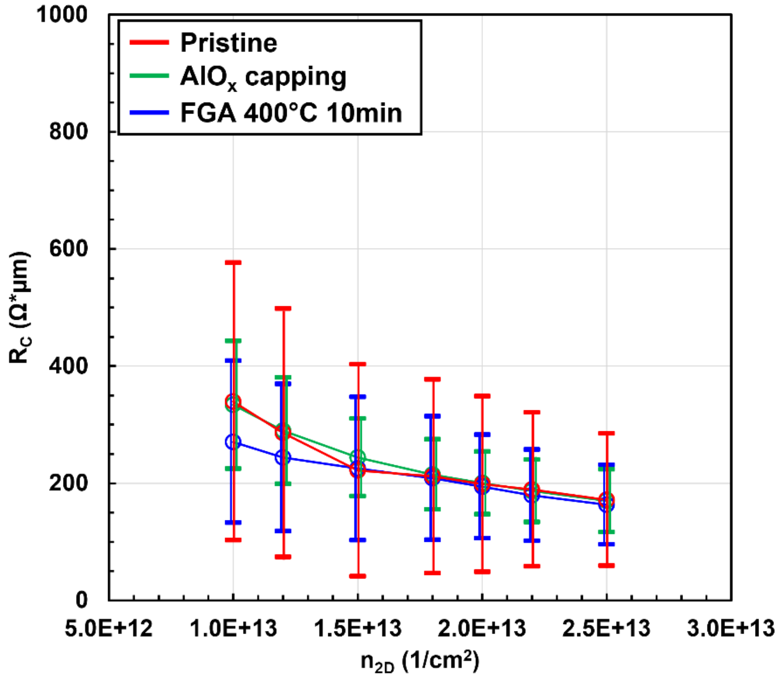

Figure S15. Statistical TLM analysis of contact resistance before and after AlO_x capping and forming gas annealing. (a) Total resistance minus metal resistance ($R_{tot} - R_{metal}$) as a function of channel length (L_{CH}) extracted from transfer characteristics at multiple carrier concentrations ranging from 2.5×10^{13} to 4×10^{12} cm^{-2} for pristine devices, devices with AlO_x capping, and devices after AlO_x capping followed by forming gas annealing (FGA, 400 °C, 10 min). The solid lines indicate linear fitting used for transmission

line method (TLM) analysis. (b) Contact resistance (R_C) extracted from statistical TLM analysis under the three processing conditions, with error bars representing device-to-device variation arising from channel-length-dependent resistance extraction. The extracted R_C values show consistently low contact resistance with limited statistical variation after AlO_x capping and subsequent FGA, indicating robust contact behavior preserved by the confinement strategy under BEOL-compatible thermal treatment.

Supplementary Materials

16. TEM/EDS line scan of pure Bi contacts in 3D monolithic structure

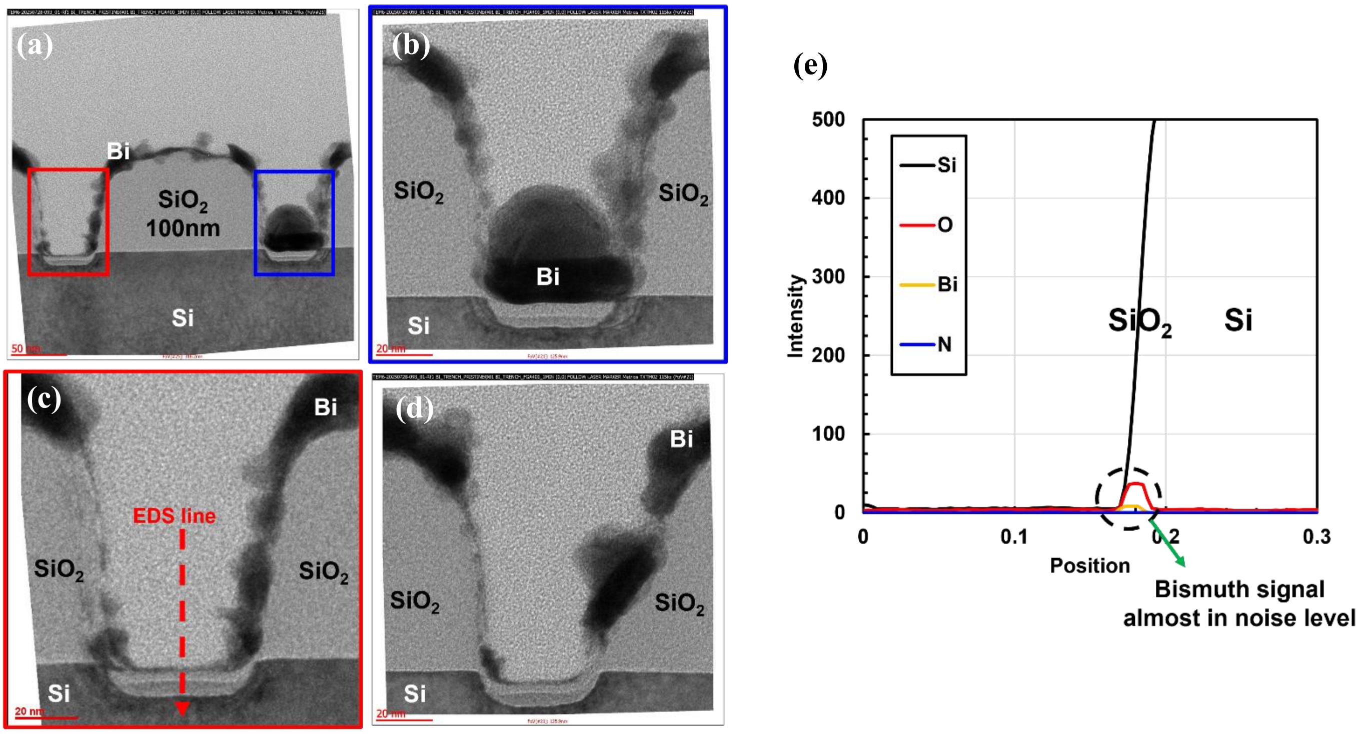

Figure S16. Bismuth contacts without confinement in a 3D monolithic structure after FGA at 400°C for 1 min. (a) Low-magnification TEM image showing two SiO_2 trenches. (b) and (c) Zoom-in TEM images of individual trenches: in (b), Bi exhibits clear outgassing, forming bumps inside the trench; in (c) and (d), Bi completely outgasses, leaving trenches devoid of metal. (e) Corresponding EDS line scan from (c), showing Bi intensity reduced to nearly noise level on the SiO_2 layer. These results provide direct evidence that low–critical-temperature semimetals such as Bi require confinement to survive BEOL annealing conditions.

Supplementary Materials

17. TEM/EDS line scan of 3D monolithic structure under different annealing environments

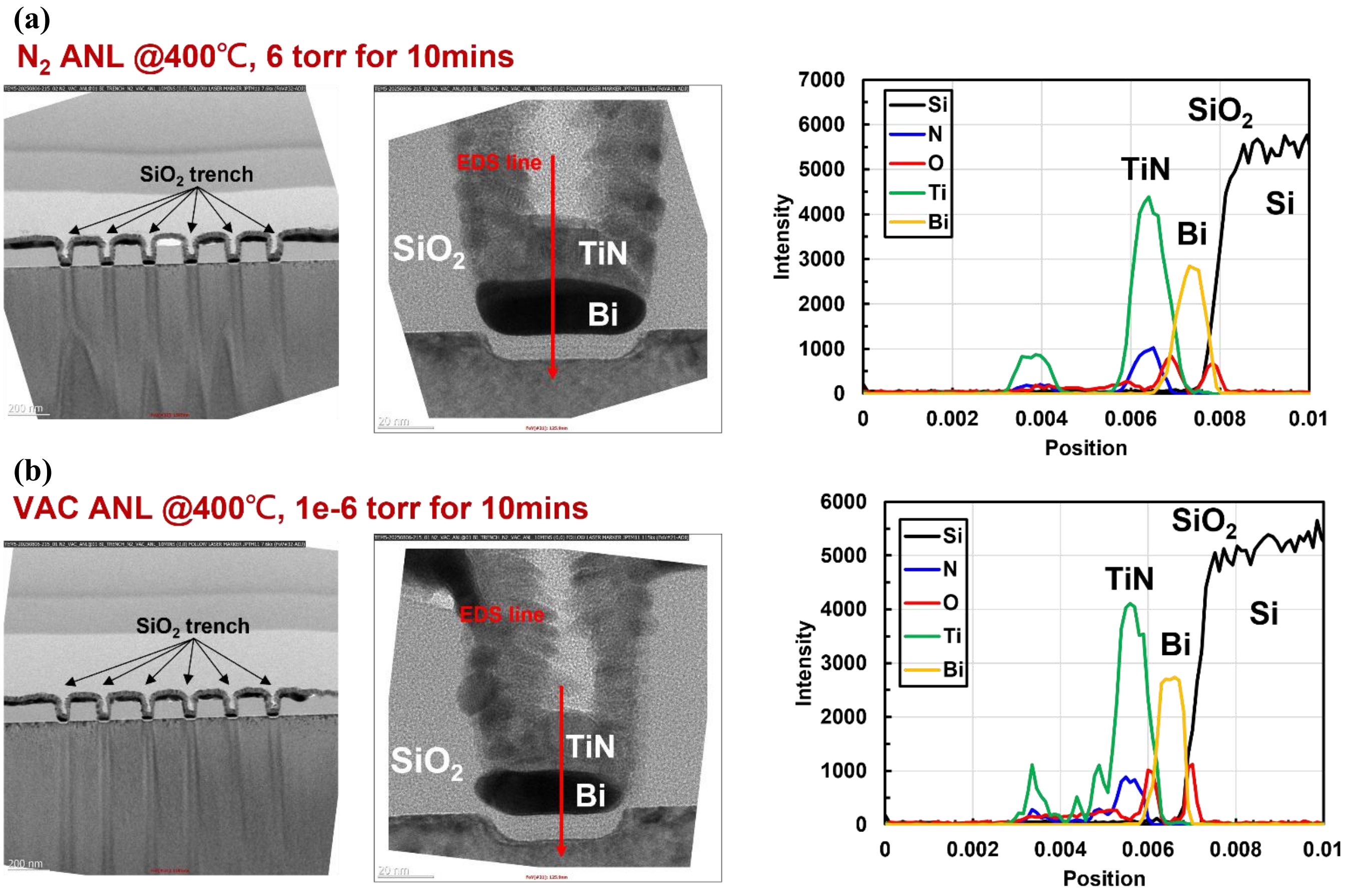

Figure S17. Bismuth contacts with TiN barrier layers in 3D monolithic structures annealed under different environments. (a) TEM and EDX analysis of six SiO_2 trenches after annealing in N_2 (400°C, 10 min), showing that Bi remains well confined under the TiN barrier without evidence of diffusion. (b) TEM and EDX analysis of six trenches annealed in high vacuum (~1 × 10^{-6} Torr, 400°C, 10 min), likewise confirming strong Bi confinement and preservation of the contact. These results demonstrate that the TiN confinement strategy is robust under multiple annealing ambients, including forming gas, N_2, and vacuum, and can sustain BEOL processing conditions.

Supplementary Materials

18. Step-by-step TEM analysis of 3D monolithic structure under extended annealing durations

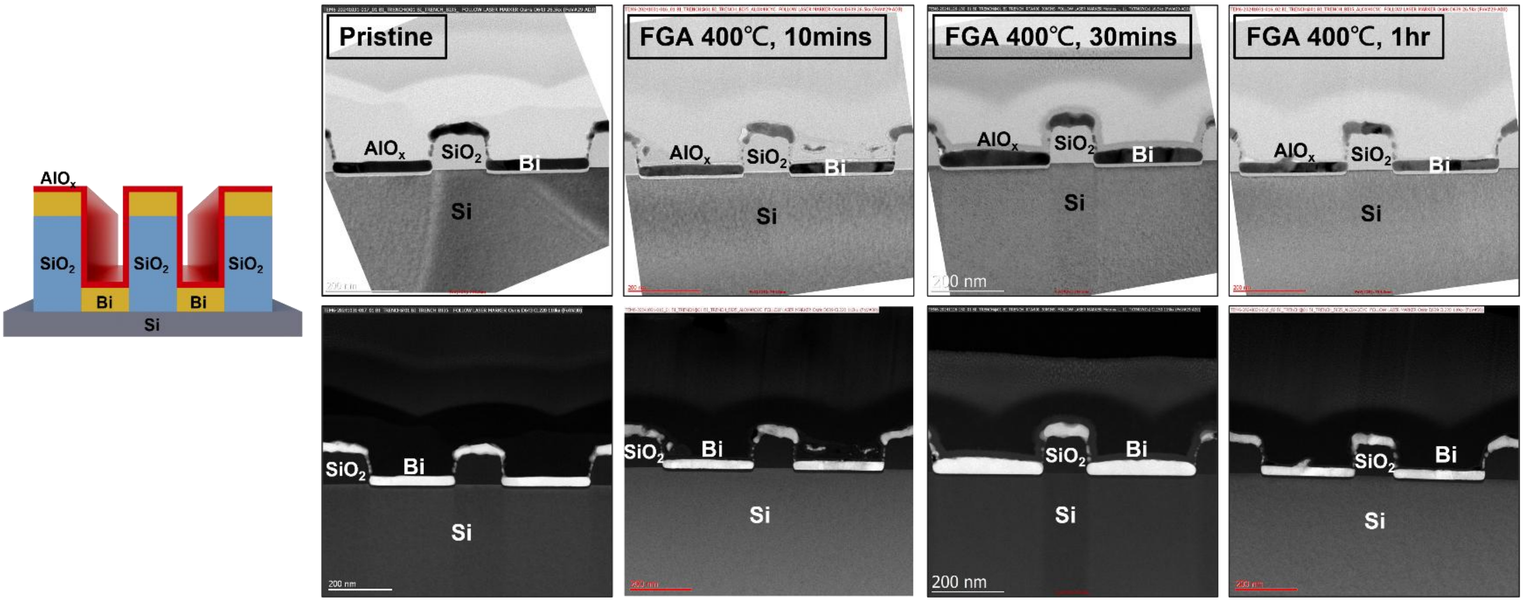

Figure S18. Stepwise TEM analysis of Bi contacts with AlO_x capping under extended BEOL annealing. TEM images (bright-field and Z-contrast) of 3D monolithic SiO_2-trench structures capped with ALD AlOx acquired at successive forming-gas annealing durations (pristine to 1 h at 400°C; 5% H_2/95% N_2) show that Bi remains intact and well confined beneath the AlO_x cap, with no evidence of outgassing, dewetting, or diffusion into the surrounding dielectric. These results confirm that AlO_x provides long-duration BEOL stability for Bi contacts, comparable to TiN-barrier confinement.

Supplementary Materials

19. Wafer-Scale Single-Crystal MoS_2 Characterization by SHG and XRD

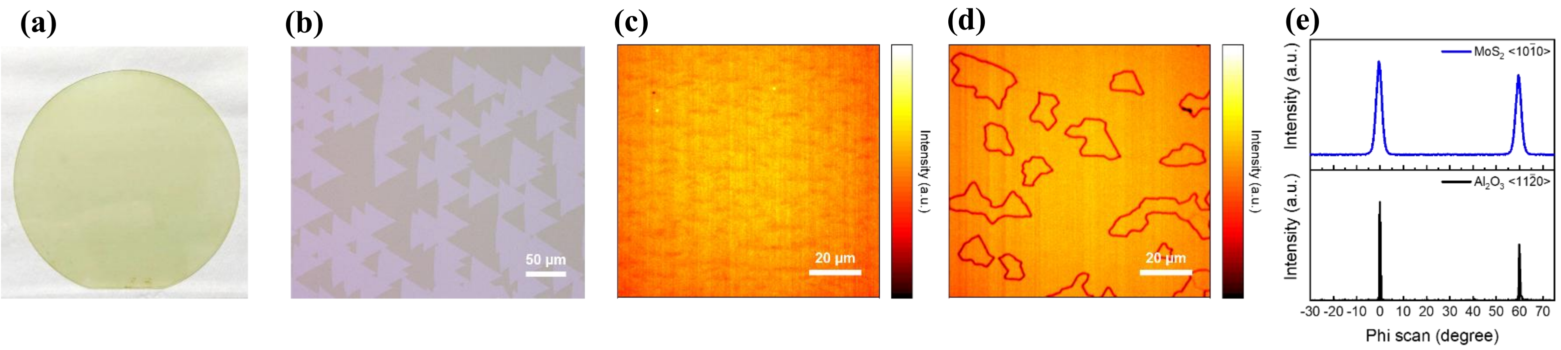

Figure S19. **Characterization of wafer-scale single-crystal MoS_2.** **(a) Photograph of the MoS_2 monolayer film grown on a 2-inch sapphire substrate. (b) Optical microscope image of individual single-crystal MoS_2 flakes. (c) Second harmonic generation (SHG) intensity map of the single-crystal MoS_2 film. The uniform SHG intensity across the scanned area demonstrates a single-crystal quality. (d) SHG intensity map of an MoS_2 film grown on an unannealed substrate. The dark lines are the grain boundaries (GBs) resulting from polycrystalline growth. (e) X-ray diffraction (XRD) Φ-scan of the MoS_2 $<10\bar{1}0>$ and sapphire $<11\bar{2}0>$ diffraction peaks.**

Supplementary Materials

Reference:

(1) Connor J. McClellan; Eilam Yalon; Kirby K. H. Smithe; Saurabh V. Suryavanshi; Eric Pop. Effective N-Type Doping of Monolayer MoS2 by AlOx. In *2017 75th Annual Device Research Conference (DRC)*; IEEE: South Bend, IN, USA, 2017.

(2) McClellan, C. J.; Yalon, E.; Smithe, K. K. H.; Suryavanshi, S. V.; Pop, E. High Current Density in Monolayer MoS2Doped by AlOx. *ACS Nano* 2021, *15* (1), 1587–1596. https://doi.org/10.1021/acsnano.0c09078.

(3) Zhang, Z.; Su, M.; Li, G.; Wang, J.; Zhang, X.; Ho, J. C.; Wang, C.; Wan, D.; Liu, X.; Liao, L. Stable Hysteresis-Free MoS2Transistors with Low-k/High-k Bilayer Gate Dielectrics. *IEEE Electron Device Letters* 2020, *41* (7), 1036–1039. https://doi.org/10.1109/LED.2020.3000259.

(4) Datye, I. M.; Gabourie, A. J.; English, C. D.; Smithe, K. K. H.; McClellan, C. J.; Wang, N. C.; Pop, E. Reduction of Hysteresis in MoS2 Transistors Using Pulsed Voltage Measurements. *2d Mater* 2019, *6* (1). https://doi.org/10.1088/2053-1583/aae6a1.

(5) Cho, K.; Park, W.; Park, J.; Jeong, H.; Jang, J.; Kim, T. Y.; Hong, W. K.; Hong, S.; Lee, T. Electric Stress-Induced Threshold Voltage Instability of Multilayer MoS2 Field Effect Transistors. *ACS Nano* 2013, *7* (9), 7751–7758. https://doi.org/10.1021/nn402348r.

(6) Illarionov, Y. Y.; Smithe, K. K. H.; Waltl, M.; Knobloch, T.; Pop, E.; Grasser, T. Improved Hysteresis and Reliability of MoS2 Transistors with High-Quality CVD Growth and Al2O3 Encapsulation. *IEEE Electron Device Letters* 2017, *38* (12), 1763–1766. https://doi.org/10.1109/LED.2017.2768602.

(7) Liu, L.; Li, T.; Ma, L.; Li, W.; Gao, S.; Sun, W.; Dong, R.; Zou, X.; Fan, D.; Shao, L.; Gu, C.; Dai, N.; Yu, Z.; Chen, X.; Tu, X.; Nie, Y.; Wang, P.; Wang, J.; Shi, Y.; Wang, X. Uniform Nucleation and Epitaxy of Bilayer Molybdenum Disulfide on Sapphire. *Nature* 2022, *605* (7908), 69–75. https://doi.org/10.1038/s41586-022-04523-5.

(8) Li, L.; Wang, Q.; Wu, F.; Xu, Q.; Tian, J.; Huang, Z.; Wang, Q.; Zhao, X.; Zhang, Q.; Fan, Q.; Li, X.; Peng, Y.; Zhang, Y.; Ji, K.; Zhi, A.; Sun, H.; Zhu, M.; Zhu, J.; Lu, N.; Lu, Y.; Wang, S.; Bai, X.; Xu, Y.; Yang, W.; Li, N.; Shi, D.; Xian, L.; Liu, K.; Du, L.; Zhang, G. Epitaxy of Wafer-Scale Single-Crystal MoS2 Monolayer via Buffer Layer Control. *Nat Commun* 2024, *15* (1). https://doi.org/10.1038/s41467-024-46170-6.

(9) Li, W.; Fan, D.; Shao, L.; Huang, F.; Liang, L.; Li, T.; Xu, Y.; Tu, X.; Wang, P.; Yu, Z.; Shi, Y.; Qiu, H.; Wang, X. High-Performance CVD MoS2Transistors with Self-Aligned Top-Gate and Bi Contact. In *Technical Digest - International Electron Devices Meeting, IEDM*; Institute of Electrical and Electronics Engineers Inc., 2021; Vol. 2021-December, pp 37.3.1-37.3.4. https://doi.org/10.1109/IEDM19574.2021.9720595.

(10) Li, T.; Guo, W.; Ma, L.; Li, W.; Yu, Z.; Han, Z.; Gao, S.; Liu, L.; Fan, D.; Wang, Z.; Yang, Y.; Lin, W.; Luo, Z.; Chen, X.; Dai, N.; Tu, X.; Pan, D.; Yao, Y.; Wang, P.; Nie, Y.; Wang, J.; Shi, Y.; Wang, X. Epitaxial Growth of Wafer-Scale Molybdenum Disulfide Semiconductor Single Crystals on Sapphire. *Nat Nanotechnol* 2021, *16* (11), 1201–1207. https://doi.org/10.1038/s41565-021-00963-8.

(11) Yu, L.; El-Damak, D.; Radhakrishna, U.; Ling, X.; Zubair, A.; Lin, Y.; Zhang, Y.; Chuang, M. H.; Lee, Y.

H.; Antoniadis, D.; Kong, J.; Chandrakasan, A.; Palacios, T. Design, Modeling, and Fabrication of Chemical Vapor Deposition Grown MoS2 Circuits with E-Mode FETs for Large-Area Electronics. *Nano Lett* 2016, *16* (10), 6349–6356. https://doi.org/10.1021/acs.nanolett.6b02739.

(12) Chris D. English; Kirby K.H. Smithe; Runjie (Lily) Xu; Eric Pop. Approaching Ballistic Transport in Monolayer MoS2 Transistors with Self-Aligned 10 Nm Top Gates. In *2016 IEEE International Electron Devices Meeting (IEDM)*; IEEE: San Francisco, CA, 2016; Vol. 10. https://doi.org/10.1109/IEDM.2016.7838355.

(13) Smithe, K. K. H.; English, C. D.; Suryavanshi, S. V.; Pop, E. Intrinsic Electrical Transport and Performance Projections of Synthetic Monolayer MoS2 Devices. *2d Mater* 2017, *4* (1). https://doi.org/10.1088/2053-1583/4/1/011009.

(14) Wu, W. C.; Hung, T. Y. T.; Sathaiya, D. M.; Chen, E.; Hsu, C. F.; Yun, W.; Hu, H. C.; Liu, B. H.; Lee, T. Y.; Kei, C. C.; Chang, W. H.; Cai, J.; Jeff, W.; Wu, C. C.; Wong, H. S. P.; Chien, C. H.; Cheng, C. C.; Radu, I. P. On the Extreme Scaling of Transistors with Monolayer MOS2 Channel. In *2024 IEEE Symposium on VLSI Technology and Circuits (VLSI Technology and Circuits)*; 2024 IEEE Symposium on VLSI Technology and Circuits (VLSI Technology and Circuits): Honolulu, HI, USA, 2024. https://doi.org/10.1109/VLSITechnologyandCir46783.2024.10631401.

(15) Chou, A. S.; Cheng, C. C.; Liew, S. L.; Ho, P. H.; Wang, S. Y.; Chang, Y. C.; Chang, C. K.; Su, Y. C.; Huang, Z. Da; Fu, F. Y.; Hsu, C. F.; Chung, Y. Y.; Chang, W. H.; Li, L. J.; Wu, C. I. High On-State Current in Chemical Vapor Deposited Monolayer MoS2nFETs with Sn Ohmic Contacts. *IEEE Electron Device Letters* 2021, *42* (2), 272–275. https://doi.org/10.1109/LED.2020.3048371.

(16) Li, W.; Gong, X.; Yu, Z.; Ma, L.; Sun, W.; Gao, S.; Köroğlu, Ç.; Wang, W.; Liu, L.; Li, T.; Ning, H.; Fan, D.; Xu, Y.; Tu, X.; Xu, T.; Sun, L.; Wang, W.; Lu, J.; Ni, Z.; Li, J.; Duan, X.; Wang, P.; Nie, Y.; Qiu, H.; Shi, Y.; Pop, E.; Wang, J.; Wang, X. Approaching the Quantum Limit in Two-Dimensional Semiconductor Contacts. *Nature* 2023, *613* (7943), 274–279. https://doi.org/10.1038/s41586-022-05431-4.

(17) Shen, P. C.; Su, C.; Lin, Y.; Chou, A. S.; Cheng, C. C.; Park, J. H.; Chiu, M. H.; Lu, A. Y.; Tang, H. L.; Tavakoli, M. M.; Pitner, G.; Ji, X.; Cai, Z.; Mao, N.; Wang, J.; Tung, V.; Li, J.; Bokor, J.; Zettl, A.; Wu, C. I.; Palacios, T.; Li, L. J.; Kong, J. Ultralow Contact Resistance between Semimetal and Monolayer Semiconductors. *Nature* 2021, *593* (7858), 211–217. https://doi.org/10.1038/s41586-021-03472-9.

(18) Kumar, A.; Schauble, K.; Neilson, K. M.; Tang, A.; Ramesh, P.; Wong, H. S. P.; Pop, E.; Saraswat, K. Sub-200 O·µm Alloyed Contacts to Synthetic Monolayer MoS2. In *Technical Digest - International Electron Devices Meeting, IEDM*; Institute of Electrical and Electronics Engineers Inc., 2021; Vol. 2021-December, pp 7.3.1-7.3.4. https://doi.org/10.1109/IEDM19574.2021.9720609.

(19) Smithe, K. K. H.; Suryavanshi, S. V.; Muñoz Rojo, M.; Tedjarati, A. D.; Pop, E. Low Variability in Synthetic Monolayer MoS2 Devices. *ACS Nano* 2017, *11* (8), 8456–8463. https://doi.org/10.1021/acsnano.7b04100.

(20) Quentin Smets; Goutham Arutchelvan; Julien Jussot; Devin Verreck; Inge Asselberghs; Ankit Nalin Mehta; Abhinav Gaur; Dennis Lin; Salim El Kazzi; Benjamin Groven; Matty Caymax; Iuliana Radu. Ultra-Scaled MOCVD MoS2 MOSFETs with 42nm Contact Pitch and 250µA/Mm Drain Current; IEEE, 2019. https://doi.org/10.1109/IEDM19573.2019.8993650.

Supplementary Materials

(21) Sebastian, A.; Pendurthi, R.; Choudhury, T. H.; Redwing, J. M.; Das, S. Benchmarking Monolayer MoS2 and WS2 Field-Effect Transistors. *Nat Commun* 2021, *12* (1). https://doi.org/10.1038/s41467-020-20732-w.

(22) Kappera, R.; Voiry, D.; Yalcin, S. E.; Branch, B.; Gupta, G.; Mohite, A. D.; Chhowalla, M. Phase-Engineered Low-Resistance Contacts for Ultrathin MoS2 Transistors. *Nat Mater* 2014, *13* (12), 1128–1134. https://doi.org/10.1038/nmat4080.

(23) Xie, L.; Liao, M.; Wang, S.; Yu, H.; Du, L.; Tang, J.; Zhao, J.; Zhang, J.; Chen, P.; Lu, X.; Wang, G.; Xie, G.; Yang, R.; Shi, D.; Zhang, G. Graphene-Contacted Ultrashort Channel Monolayer MoS2 Transistors. *Advanced Materials* 2017, *29* (37). https://doi.org/10.1002/adma.201702522.

(24) Rai, A.; Valsaraj, A.; Movva, H. C. P.; Roy, A.; Ghosh, R.; Sonde, S.; Kang, S.; Chang, J.; Trivedi, T.; Dey, R.; Guchhait, S.; Larentis, S.; Register, L. F.; Tutuc, E.; Banerjee, S. K. Air Stable Doping and Intrinsic Mobility Enhancement in Monolayer Molybdenum Disulfide by Amorphous Titanium Suboxide Encapsulation. *Nano Lett* 2015, *15* (7), 4329–4336. https://doi.org/10.1021/acs.nanolett.5b00314.

(25) Lu, H.; Kummel, A.; Robertson, J. Passivating the Sulfur Vacancy in Monolayer MoS2. *APL Mater* 2018, *6* (6). https://doi.org/10.1063/1.5030737.

(26) Bretscher, H.; Li, Z.; Xiao, J.; Qiu, D. Y.; Refaely-Abramson, S.; Alexander-Webber, J. A.; Tanoh, A.; Fan, Y.; Delport, G.; Williams, C. A.; Stranks, S. D.; Hofmann, S.; Neaton, J. B.; Louie, S. G.; Rao, A. Rational Passivation of Sulfur Vacancy Defects in Two-Dimensional Transition Metal Dichalcogenides. *ACS Nano* 2021, *15* (5), 8780–8789. https://doi.org/10.1021/acsnano.1c01220.

(27) Kim, S. Y.; Park, S.; Choi, W. Enhanced Carrier Mobility of Multilayer MoS2 Thin-Film Transistors by Al2O3 Encapsulation. *Appl Phys Lett* 2016, *109* (15). https://doi.org/10.1063/1.4964606.

(28) Radisavljevic, B.; Kis, A. Mobility Engineering and a Metal-Insulator Transition in Monolayer MoS 2. *Nat Mater* 2013, *12* (9), 815–820. https://doi.org/10.1038/nmat3687.
